# TREM2 deficiency aggravates renal injury by promoting macrophage apoptosis and polarization via the JAK-STAT pathway in mice

**DOI:** 10.1038/s41419-024-06756-w

**Published:** 2024-06-07

**Authors:** Yan Cui, Chao Chen, Zhouqi Tang, Wenjia Yuan, Kaiye Yue, Pengcheng Cui, Xia Qiu, Hedong Zhang, Tengfang Li, Xuejing Zhu, Jiadi Luo, Siyu Sun, Yaguang Li, Chen Feng, Longkai Peng, Xubiao Xie, Yong Guo, Yixin Xie, Xin Jiang, Zhongquan Qi, Angus W. Thomson, Helong Dai

**Affiliations:** 1 Medical College, Guangxi University, Nanning, 530004 China; 2 Department of Kidney Transplantation, Center of Organ Transplantation, The Second Xiangya Hospital of Central South University, Changsha, Hunan 410011 China; 3 Department of Nephrology, Hunan Key Laboratory of Kidney Disease and Blood Purification, The Second Xiangya Hospital of Central South University, Changsha, Hunan 410011 China; 4 Department of Pathology, The Second Xiangya Hospital of Central South University, Changsha, Hunan 410011 China; 5 Department of Organ Transplantation, The Fifth Clinical Medical College of Henan University of Chinese Medicine (Zhengzhou People’s Hospital), Zhengzhou, Henan 450000 China; 6 Starzl Transplantation Institute, Department of Surgery and Department of Immunology, University of Pittsburgh School of Medicine, Pittsburgh, PA 15213 USA

**Keywords:** Mechanisms of disease, Cell death and immune response

## Abstract

The triggering receptor expressed on myeloid cells 2 (TREM2) is an immune receptor that affects cellular phenotypes by modulating phagocytosis and metabolism, promoting cell survival, and counteracting inflammation. Its role in renal injury, in particular, unilateral ureteral obstruction (UUO) or ischemia-reperfusion injury (IRI)-induced renal injury remains unclear. In our study, WT and *Trem2*^−/−^ mice were employed to evaluate the role of TREM2 in renal macrophage infiltration and tissue injury after UUO. Bone marrow-derived macrophages (BMDM) from both mouse genotypes were cultured and polarized for in vitro experiments. Next, the effects of TREM2 on renal injury and macrophage polarization in IRI mice were also explored. We found that TREM2 expression was upregulated in the obstructed kidneys. TREM2 deficiency exacerbated renal inflammation and fibrosis 3 and 7 days after UUO, in association with reduced macrophage infiltration. *Trem2*^−/−^ BMDM exhibited increased apoptosis and poorer survival compared with WT BMDM. Meanwhile, TREM2 deficiency augmented M1 and M2 polarization after UUO. Consistent with the in vivo observations, TREM2 deficiency led to increased polarization of BMDM towards the M1 proinflammatory phenotype. Mechanistically, TREM2 deficiency promoted M1 and M2 polarization via the JAK-STAT pathway in the presence of TGF-β1, thereby affecting cell survival by regulating mTOR signaling. Furthermore, cyclocreatine supplementation alleviated cell death caused by TREM2 deficiency. Additionally, we found that TREM2 deficiency promoted renal injury, fibrosis, and macrophage polarization in IRI mice. The current data suggest that TREM2 deficiency aggravates renal injury by promoting macrophage apoptosis and polarization via the JAK-STAT pathway. These findings have implications for the role of TREM2 in the regulation of renal injury that justify further evaluation.

## Introduction

Inflammation and fibrosis are common pathological manifestations of progressive, chronic kidney disease (CKD) that eventually result in end-stage renal disease [1]. It has been documented that prolonged and excessive inflammation plays a significant role in the development of fibrosis [2, 3]. The pathogenesis of the unilateral ureteral obstruction (UUO) animal model mirrors that of human obstructive nephropathy. Specifically, complete blockage of the renal system leads to damage of tubular epithelial cells, resulting in a notable increase in markers of renal fibrosis, such as inflammation mediated by macrophages and activation of myofibroblasts [4]. Furthermore, renal IRI involves a sequence where the kidney experiences an ischemic phase followed by reperfusion under specific conditions. Despite reperfusion, renal function does not recover but rather exacerbates the damage. The pathophysiology of IRI is intricate, encompassing various factors that contribute to kidney injury and fibrosis [5]. Additionally, an increasing number of studies have acknowledged the key and complex role of macrophages in renal inflammation and fibrosis [6, 7]. Macrophage plasticity plays an important role in the process from renal inflammation to renal fibrosis, which is an important step in the development of end-stage renal disease. On one end of the spectrum, M1 macrophages, which are proinflammatory, play a role in clearing infections but can also exacerbate renal damage. Conversely, M2 macrophages, which are anti-inflammatory, possess a reparative phenotype but also exacerbate renal fibrosis [8]. Despite this understanding, the precise mechanisms involved in these processes are not fully elucidated, and effective treatments are currently lacking.

Triggering receptor expressed on myeloid cells 2 (TREM2) is an immune receptor expressed on myeloid cells, including immature dendritic cells [9], osteoblasts [10], microglia [11, 12], and other macrophages [13, 14]. It consists of an extracellular domain that includes a single V-type immunoglobulin domain, a short ectodomain, a single transmembrane helix, and a short cytosolic tail that lacks any signal transduction or trafficking motifs [15]. TREM2 binds to the junctional proteins DNAX activation protein 10 (DAP10) or DAP12, resulting in the activation of phosphoinositide 3-kinase (PI3K) or spleen tyrosine kinase (Syk), which in turn mediate downstream signaling [16]. Furthermore, TREM2 has been reported to regulate inflammatory signaling and microglial metabolism and to promote microglial phagocytosis, activation, survival, and proliferation [17, 18]. Most studies on TREM2 have focused on microglia in the brain. However, the role of TREM2 in liver and lung injury has also been studied in recent years. For example, Perugorria et al. reported that *Trem2*^−/−^ mice exhibited more severe liver injury and inflammation during acute and chronic CCl_4_ and acetaminophen intoxication [19]. Moreover, Vincent et al. observed that *Trem2*^−/−^ mice experienced worse LPS-induced acute lung injury [20], while Nakao et al. documented more severe liver ischemia-reperfusion injury (IRI) in *Trem2*^−/−^ mice [21]. Nevertheless, the influence of TREM2 on macrophages and underlying mechanisms during the progression of renal injury/fibrosis remains unclear.

In this study using a mouse model of unilateral ureteral obstruction (UUO), we observed a significant increase in expression of TREM2 with duration of disease in injured kidneys compared to sham controls. Consistent with our hypothesis, renal inflammation and fibrosis were exacerbated in UUO mice lacking TREM2. This was accompanied by a decrease in kidney-infiltrating macrophages, while the incidence of M1 and M2 cells increased. In vitro experiments revealed that TREM2 deficiency resulted in the downregulation of the mammalian target of rapamycin (mTOR) signaling and induced macrophage apoptosis. Conversely, TREM2 deficiency triggered activation of the JAK-STAT pathway to promote macrophage polarization towards M1 and M2. Furthermore, the data from another renal fibrosis mouse model, IRI, had consistent results. We demonstrated that TREM2 significantly improved IRI- and UUO-induced kidney injury by alleviating inflammation and fibrosis, and potently abrogated the transition to CKD.

## Methods and materials

### Animal experiments

WT mice used in these experiments were male C57BL/6J mice aged 8 to 12 weeks and were purchased from SLAC Jingda Laboratory Animal Co. Ltd (Hunan, China). *Trem2* knockout (*Trem2*^*−/−*^) mice were generated using conventional homologous recombination methods by Cyagen Biosciences, Inc. (Suzhou, China). The offspring were genotyped using PCR according to the manufacturer’s protocol (Cyagen, Suzhou, China). The UUO and IRI model were established as previously described [22, 23]. The UUO mouse models were euthanized on day 3 or day 7 after the UUO or sham operation. The IRI mice models were euthanized on day 7. Kidney tissues were harvested for various molecular and histological studies.

### RNA extraction and quantitative real-time PCR (qRT-PCR)

Kidney tissue and cell pellets were harvested. Total RNA was extracted using TRIzol reagent according to the manufacturer’s instructions (Vazyme, Nanjing, China), and cDNA was synthesized using the reverse transcription system kits (Vazyme). Real-time PCR was performed using the SYBR Green qPCR Master Mix kit (Bimake, Houston, TX, USA). Primer sequences for the analyzed genes are listed in Table S1. Quantitative PCR data were analyzed using the 2^−∆∆ct^ method.

### Immunofluorescence (IF) staining

For IF studies, kidney tissues were embedded at the optimal cutting temperature (OCT) immediately after the mice were euthanized. OCT-embedded kidneys were cryosectioned into 5-μm sections and mounted on adhesion slides. The sections were fixed with cold methanol for 20 min, blocked with 5% v/v goat serum for 1 h at room temperature (RT), and incubated with primary antibodies against TREM2 (Proteintech, Wuhan, China), F4/80 (Servicebio, Wuhan, China), kidney injury molecule-1 (KIM-1; Novus, Port Orchard, WA, USA), inducible nitric oxide synthase (iNOS; Servicebio), CD206 (Servicebio), and Arg1 (Servicebio). The secondary antibodies used were Alexa Fluor 488 and Alexa Fluor 594 (Servicebio). The sections were then stained with 40, 6-diamidino-2phenylindole. All images were captured using a confocal microscope (ECLIPSE Ti; Nikon, Tokyo, Japan).

### Kidney histology

The kidneys embedded in paraffin were sectioned at 5 μm thickness and stained with hematoxylin and eosin using standard methods. Indices of tubular damage (tubular dilation, cell necrosis, infarction, and cast formation) were assessed by calculating the percentage of the renal cortex that displayed these features [24]. Histological assessment was performed in a blinded fashion in five randomly selected fields (original magnification × 100).

### TdT-mediated dUTP nick end labeling (TUNEL) staining

TUNEL staining to detect cell death was performed using a commercially available in situ cell death detection kit (Servicebio) according to the manufacturer’s instructions.

### Immunohistochemistry and Masson staining

The kidney tissues were fixed in 4% v/v paraformaldehyde for 48 h, dehydrated using a graded ethanol series, embedded in paraffin, and sectioned (5 μm). For immunohistochemical staining, antigen retrieval was performed using a citrate solution after deparaffinization and rehydration. The sections were blocked with 5% v/v goat serum and then incubated with the following primary antibodies at 4 °C overnight: collagen-I (col-I; Abcam, Cambridge, England) and α-smooth muscle actin (α-SMA; Abcam). The sections were then incubated with biotinylated secondary antibodies (Abcam) for 2 h at RT and streptavidin-peroxidase complex for 1 h at RT then visualized with a 3,3′-diaminobenzidine system according to the manufacturer’s protocol. The sections were counterstained with hematoxylin and dehydrated. The dehydrated sections were mounted using neutral resin. Masson’s trichrome staining was performed according to the manufacturer’s instructions (Servicebio). Quantification of collagen-I-, α-SMA-, and Masson-positive areas was performed by taking random cortical images (original magnification power × 40, six fields per kidney) of renal cortex from each mouse and counting the percentages of the positively-stained areas in each microscopic field.

### Cell culture and treatment

Murine bone marrow-derived macrophages (BMDM) were generated using a procedure similar to that described previously [25, 26]. Briefly, the BM was isolated from the tibias and femurs and cultured in DMEM (CGM102.05, Cellmax, Beijing, China.) containing 10% v/v fetal bovine serum (FBS; 10100147, Gibco, Grand Island, NY, USA), 20 ng/mL macrophage colony-stimulating factor (M-CSF; R&D Systems, Minneapolis, MN, USA), 100 U/mL penicillin, and 100 μg/mL streptomycin. The cells were allowed to differentiate into macrophages for 7 days. On day 7, BMDMs were cultured for 24 h in complete media alone (M0) or media supplemented with 50 ng/mL LPS (Sigma-Aldrich, St. Louis, MO, USA), 10 ng/mL IL-4 (PeproTech, Rocky Hill, NJ, USA) or 5 ng/mL TGF-β1 (R&D Systems). After incubation, cells were washed with sterile PBS and used for experimental assays.

### Tissue harvest for single-cell suspension and flow cytometric analysis

Single-cell suspensions were isolated from murine kidneys by digestion with collagenase and DNaseI. Erythrocytes were lysed using an ammonium chloride potassium lysis buffer. The cells were collected by centrifugation (500 × *g* for 5 min at RT) and washed with phosphate-buffered saline containing 2% v/v FBS. The cells were stained with Zombie Aqua (BioLegend, San Diego, CA) and treated with FcγR-blocking rat anti-mouse CD16/32 Ab (Cat# 101320; BioLegend). For surface staining, cells were incubated for 30 min at 4 °C with different combinations of fluorochrome-conjugated Abs, including CD45.2 (Cat# 103116), CD11b (Cat# 101216), F4/80 (Cat# 123107), CD86 (Cat# 105028) and major histocompatibility complex class II (MHC II, CD274; 10F.9G2, BioLegend). For intracellular staining, cells were fixed and permeabilized using a Fixation and Permeabilization reagent (BioLegend), followed by incubation with CD206 (Cat# 141708, BioLegend). All flow cytometry data were acquired using an LSR-Fortessa flow cytometer (BD Biosciences, San Jose, CA, USA) and analyzed using FlowJo version 10 software (Tree Star, San Carlos, CA).

### Cell counting kit-8 (CCK-8) assay

Cells were seeded in 96-well culture plates (1 × 10^5^/well) and incubated at 37 °C in a 5% CO_2_ incubator. The old solution was discarded on days 1–7 and 95 μL of RPMI-1640 medium and 5 μL of CCK-8 was added to each well of the 96-well plate to be tested, then incubated at 37 °C for 2 h. A microplate reader (Thermo Fisher Scientific, Waltham, MA, USA) was used to measure the optical density (OD) of each well at 450 nm and to detect changes in the cell proliferation ability of each group. In the 1-carboxymethyl-2-iminoimidazolidine (cyclocreatine) experiments, 10 nM cyclocreatine was added on days 3, 4, and 5 to the BM cell culture.

### Western blotting

BMDMs were lysed in RIPA buffer containing a protease and phosphatase inhibitor cocktail (Coolabor, Beijing, China). Protein concentration was determined using the BCA assay (Vazyme). Equal amounts of protein were loaded onto a 4–12% sodium dodecyl sulfate-polyacrylamide gel and transferred to a polyvinylidene difluoride membrane (GE Healthcare, Little Chalfont, Buckinghamshire, UK). The membrane was blocked with 5% v/v milk and incubated overnight at 4 °C with the primary antibodies (listed in Table S2). After the membranes were washed with TBST buffer 3 times, they were probed with secondary antibodies (Antgene, Wuhan, China) for 1 h at RT and then visualized using an enhanced chemiluminescence kit (Advance Targeting System, San Diego, CA, USA).

### RNA-seq

Transcriptome sequencing of BMDM was performed by GENE DENOVO Co. Ltd (Guangzhou, China). Genes with significantly different expression levels (upregulated by at least twofold or downregulated by at least 0.5-fold) in *Trem2*^−/−^ BMDM compared to WT BMDM were selected for further analysis. Genes that were commonly upregulated or downregulated in *Trem2*^−/−^ BMDM were identified and analyzed using the KEGG pathway database (http://www.genome.jp/kegg/). Hierarchical clustering of differential gene expression patterns was performed and heat maps were used to present the clustering results. Subsequently, GSEA analysis of the KEGG pathway was performed.

### Statistical analyses

All data were presented as mean ± standard deviation (SD) values and tested for normal distribution. Statistical analyses were conducted using GraphPad Prism 8.0. The *t*-test was utilized to compare two groups, while the one-way analysis of variance (ANOVA) with post hoc Tukey multiple comparison tests was used to compare more than two groups. *P* < 0.05 was considered statistically significant.

## Results

### TREM2 was upregulated in the obstructed kidney

**Fig. 1:**
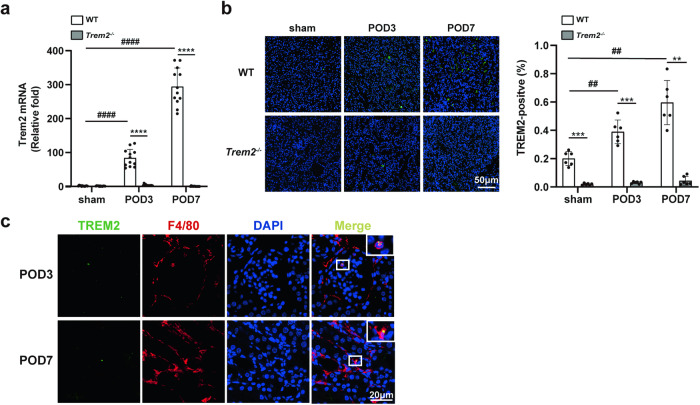
Augmented TREM2 expression is observed in UUO kidneys and TREM2 is partially co-expressed with F4/80. A UUO mouse model was established. TREM2 expression in kidneys was assessed by **a** qRT-PCR (*n* = 12) and **b** IF staining (*n* = 6). **c** Co-expression of TREM2 and F4/80 in UUO kidneys of WT mice on POD3 and 7 was detected by IF double-labeling. Green: TREM2; red: F4/80; blue: DAPI. **P < 0.01, ***P < 0.001, ****P < 0.0001 vs. sham group; ^##^*P* < 0.01, ^####^*P* < 0.0001 vs. WT group. POD postoperative day.

First, we investigated the expression of TREM2 in UUO kidneys. While qPCR analysis did not detect Trem2 mRNA in sham kidneys, it was significantly upregulated in obstructed kidneys (Fig. 1a). Indeed, Trem2 mRNA levels in obstructed kidneys on postoperative day (POD) 7 were significantly upregulated than that observed in the sham group. Additionally, IF staining of TREM2 in the kidneys demonstrated a similar pattern (Fig. 1b). Subsequently, to ascertain the cellular localization of TREM2 in the kidneys, double IF staining was conducted. The findings indicated a strong co-localization of TREM2 and the macrophage marker F4/80 (Fig. 1c), suggesting that macrophages in the kidney are the predominant cell type that expresses TREM2, consistent with previous reports [15, 27, 28]. These findings suggest that TREM2 is a crucial regulator in UUO progression and deserves our further investigation.

### TREM2 deficiency aggravated UUO-induced renal injury, renal cell apoptosis, and inflammation

**Fig. 2:**
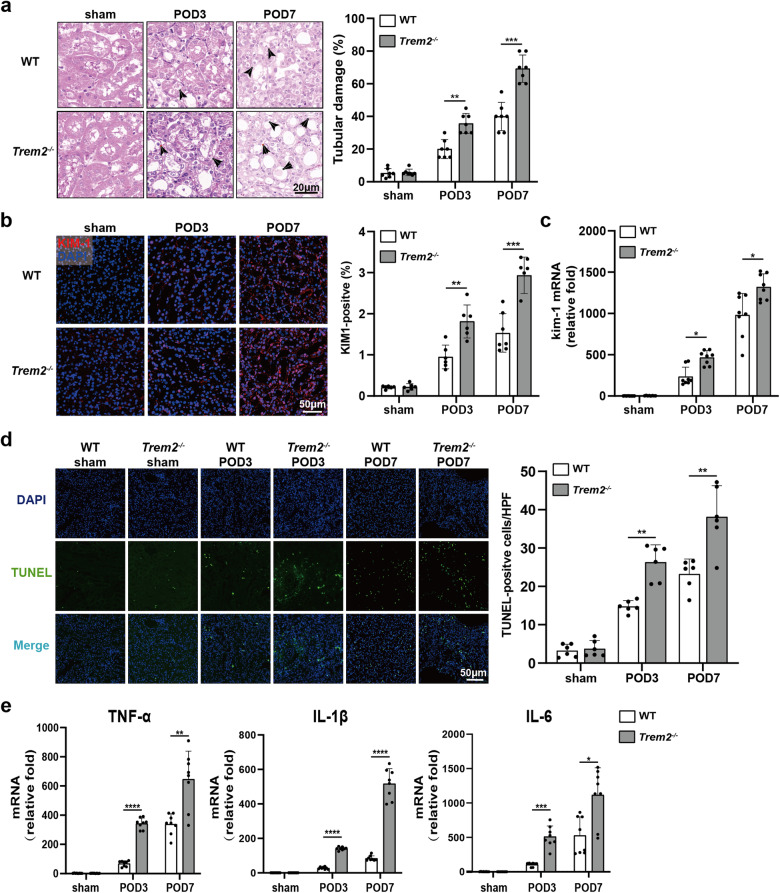
TREM2 deficiency aggravates tubular injury, renal apoptosis, and renal inflammation in UUO mice. **a** Left; H&E staining of UUO kidneys from WT and *Trem2*^−/−^ mice (*n* = 7). Black arrows point to damage to kidney tissue (vacuolation of cells, loss of brush border, tubular formation, etc.), right; H&E results were assessed and quantified as the percentage of the tubular damage area. **b** Kim-1 expression levels in the kidneys of WT and *Trem2*^−/−^ mice by IF (*n* = 6) and **c** RT-PCR (*n* = 7). Red: KIM-1; blue: DAPI. Quantitative analysis of KIM-1 IF results by Image Pro Plus 6.0 software. **d** TUNEL staining of UUO kidney tissues from both groups of mice; TUNEL-positive cells were manually counted, *n* = 6 per group. Green: TUNEL, blue: DAPI. **e** TNF-α, IL-1β, and IL-6 mRNA levels were measured by RT-PCR in the UUO kidneys of WT and *Trem2*^−/−^ mice, *n* = 8 per group. *P < 0.05, **P < 0.01, ***P < 0.001, ****P < 0.0001.

Next, the effect of TREM2 on renal injury induced by UUO was explored. *Trem2*^*−/−*^ mice exhibited significant exacerbation of UUO-induced tubular damage, characterized by cortical tubular dilation accompanied by tubular epithelial cell necrosis, brush border loss, intratubular cast formation, and immune cell infiltration (Fig. 2a). However, no significant difference was observed in the levels of serum creatinine (Cr) and blood urea nitrogen (BUN) between WT and *Trem2*^*−/−*^ mice on POD3, 7, and 14, compared with the sham group (Fig. S1). In addition, TREM2 deficiency had no significant effect on systemic immune infiltration and inflammatory response (Fig. S2). This may be due to an existing compensatory functional role of the contralateral healthy kidney [29, 30]. Therefore, we examined the expression of KIM-1, an important molecular indicator of renal injury [31]. Both qRT-PCR and IF results revealed that the KIM-1 expression level was significantly higher in the UUO kidneys of *Trem2*^*−/−*^ mice compared with the UUO kidneys of WT mice (Fig. 2b, c).

Moreover, renal apoptosis plays a crucial role in the pathogenesis of UUO [32]. In the current study, TUNEL analysis indicated that the renal cell apoptosis induced by UUO was significantly increased in *Trem2*^*−/−*^ mice (Fig. 2d), indicating that TREM2 deficiency led to increased apoptosis in the UUO kidneys. Furthermore, inflammatory cytokines, including TNF-α, IL-1β, and IL-6, are produced and secreted by inflammatory cells, such as macrophages, during UUO progression and can reflect the level of inflammation [33, 34]. As expected, on POD3 and 7, TNF-α, IL-1β, and IL-6 mRNA levels were significantly higher in the UUO kidneys of *Trem2*^*−/−*^ compared with WT mice (Fig. 2e). Overall, the above data indicate that TREM2 deficiency significantly exacerbates tubular injury, renal inflammation, and renal cell apoptosis in UUO kidneys.

### TREM2 deficiency aggravated UUO-induced renal fibrosis

**Fig. 3:**
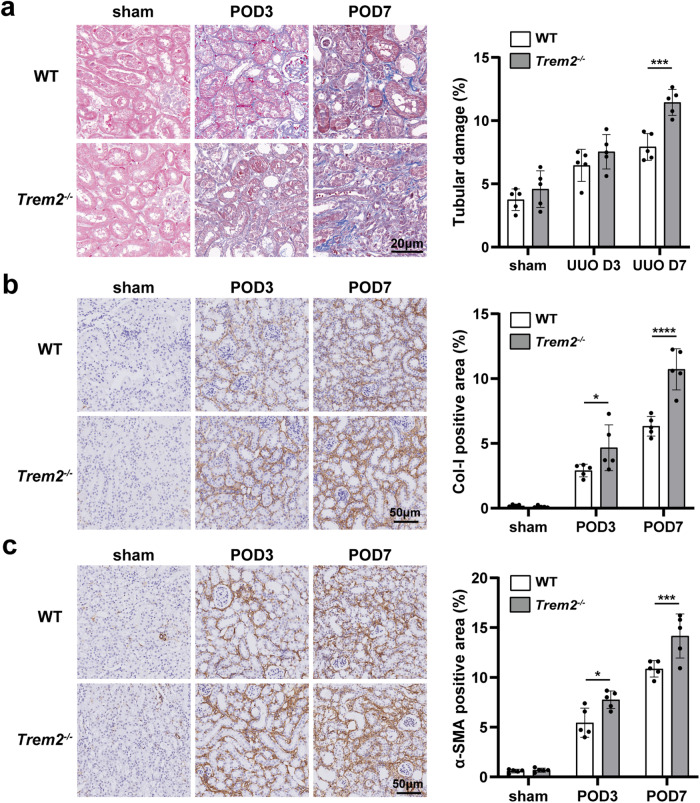
TREM2 deficiency increases interstitial fibrosis in the kidney after UUO in mice. **a** Masson staining of UUO kidney tissues from WT and *Trem2*^−/−^ mice; results were quantified by Image Pro Plus 6.0 software to assess the level of renal fibrosis in mice. Immunohistochemical labeling and quantitative analyses for col-I (**b**) and α-SMA (**c**) in UUO kidneys showed more renal interstitial fibrosis. *n* = 5 per group. *P < 0.05, ***P < 0.001, ****P < 0.0001.

Blocked urinary flow upon UUO leads to tubular injury, which in turn, leads to renal fibrosis [35]. To further investigate the influence of TREM2 on kidney fibrosis in mice with UUO, we performed Masson staining on UUO kidneys of both WT and *Trem2*^*−/−*^ mice. A significant increase in the extent of fibrosis was then observed in *Trem2*^*−/−*^ mice on POD7 (Fig. 3a). In addition, immunohistochemical analysis was performed to assess the expression levels of col-I and α-SMA, classic profibrotic markers, in the UUO kidneys of WT and *Trem2*^*−/−*^ mice. Significant increases in the levels of col-I and α-SMA were observed (Fig. 3b, c). Taken together, these results indicate that TREM2 deficiency significantly exacerbates renal fibrosis following UUO.

### TREM2 deficiency inhibited macrophage survival in UUO kidneys and BMDMs

**Fig. 4:**
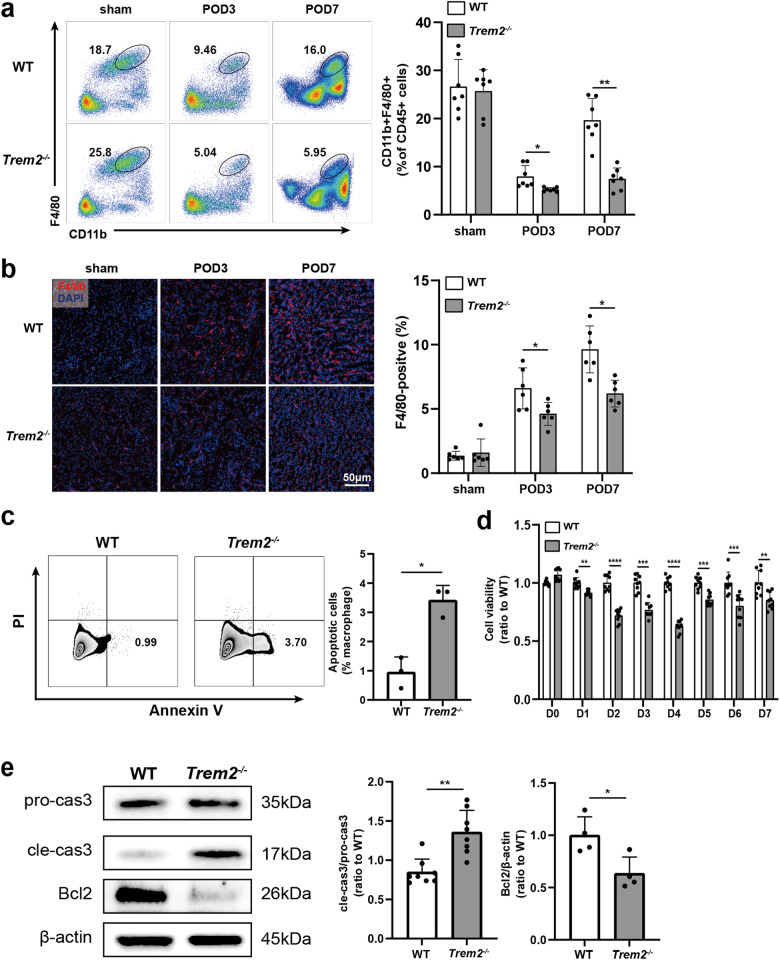
TREM2 deficiency inhibits macrophage survival in vivo and in vitro. Assessment of the proportion of infiltrating macrophages (CD11b^+^ F4/80^+^) in UUO kidneys of two groups of mice (gated on CD45^+^ cells) by **a** Flow cytometry (*n* = 7) and **b** IF staining (*n* = 6). **c** WT, *Trem2*^−/−^ BMDM for Annexin V-PI apoptosis detection by Flow Cytometry, *n* = 3 per group. Annexin V^+^ PI^−^ represents early apoptosis, while Annexin V^+^ PI^+^ represents late apoptosis. (**d**) WT, *Trem2*^−/−^ BMDM in vitro culture day 0-7 for CCK-8 results (WT BMDM as control), *n* = 9 per group. **e** Western blotting assay of pro-caspase 3 (*n* = 8), cleaved-caspase 3 (*n* = 8), and Bcl2 (*n* = 4) proteins in WT, *Trem2*^−/−^ BMDM. *P < 0.05, **P < 0.01, ***P < 0.001, ****P < 0.0001.

Extensive research has consistently demonstrated that renal inflammatory cell infiltration, particularly the infiltration of macrophages, plays a prominent role in UUO pathogenesis [36, 37]. To explore the mechanism underlying the regulation of UUO macrophages by TREM2, we assessed infiltrating macrophages (CD11b^+^ F4/80^+^) within the UUO kidneys. The results indicate a significant decrease in the proportion of infiltrating macrophages in UUO kidneys of *Trem2*^−/−^ compared to WT mice on POD3, with a further reduction on POD7 (Fig. 4a, gating strategy provided in Figure S3). Similarly, IF staining revealed that the number of F4/80^+^ cells (macrophages) infiltrating UUO kidneys of *Trem2*^*−/−*^ mice was significantly reduced on POD3 and POD7 (Fig. 4b). These data indicate that TREM2 deficiency markedly attenuated macrophage infiltration in UUO kidneys.

To further ascertain the influence of TREM2 on macrophage development/survival in vitro, we assessed apoptosis/survival of BMDM on day 5 of culture. The results show that TREM2 deficiency significantly increased the incidence of early apoptotic cells (Annexin V^+^ PI^−^) in BMDM (Fig. 4c). Moreover, the CCK-8 assay demonstrated that TREM2 deficiency significantly inhibited cell survival during the development of BM cells into macrophages, and that the difference was most significant on day 4 (Fig. 4d). Furthermore, immunoblotting results indicated that anti-apoptotic protein Bcl2 was significantly downregulated in *Trem2*^*−/−*^ BMDM, while the cleaved-caspase 3/pro-caspase 3 level was upregulated (Fig. 4e), confirming that apoptosis was enhanced in *Trem2*^*−/−*^ BMDM compared to WT BMDM. Collectively, these data suggest that TREM2 deficiency promotes macrophage apoptosis and inhibits its survival.

### TREM2 deficiency impaired mTOR signaling in BMDM

**Fig. 5:**
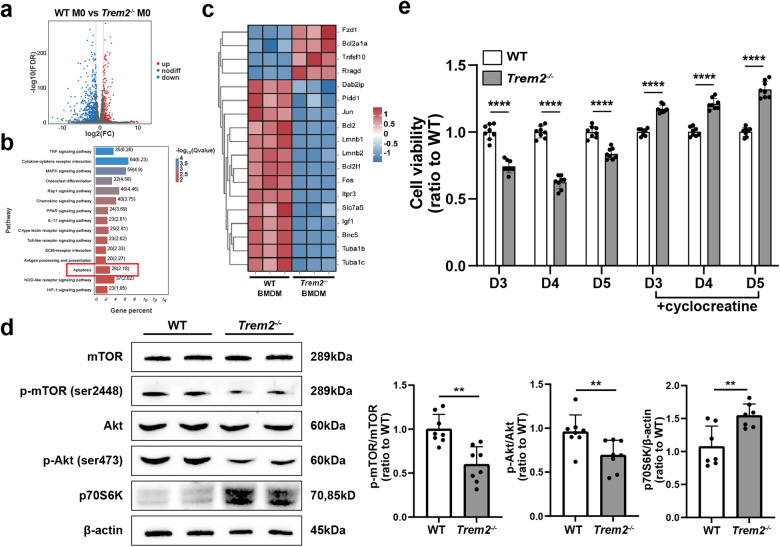
TREM2 regulates macrophage survival through the Akt/mTOR pathway. **a** Volcano plot for WT, *Trem2*^−/−^ BMDM difference comparison. The horizontal coordinates indicate the logarithmic value of the difference in ploidy between the two subgroups, and the vertical coordinates indicate the negative Log10 value of the FDR of the difference between the two subgroups. Red (*Trem2*^−/−^ upregulated relative to WT expression) and blue (expression downregulated) points indicate a significant difference in gene expression (judged by FDR < 0.05, and more than two-fold difference), and black points indicate a lack of difference. **b** WT, *Trem2*^−/−^ KEGG enrichment bar graph of differential genes in BMDM. The vertical coordinate represents each signaling pathway, the horizontal coordinate represents the number of that signaling pathway as a percentage of the number of all differential genes, the darker the color the smaller the *Q* value, and the value on the bar represents the number of that signaling pathway and the *Q* value. **c** Heatmap of WT, *Trem2*^−/−^ BMDM for differential comparative clustering analysis. Each column in the graph represents a sample, each row represents a gene, and the expression of genes in different samples is indicated by different colors, with red colors indicating higher expression and blue colors indicating lower expression. **d** The differences in expression levels of mTOR, p-mTOR (ser2448), Akt, p-Akt (ser473), and p70S6K, key proteins of the mTOR signaling pathway, were detected by western blotting, and the bands analyzed in grayscale and statistically by using ImageJ software, *n* = 7–8. **e** Comparison of the CCK-8 results of WT and *Trem2*^−/−^ BMDM in vitro cultures after the addition of cyclocreatine days 3–5, *n* = 8. **P < 0.01, ****P < 0.0001.

In order to explore the specific mechanisms by which TREM2 acts on macrophages, we performed transcriptomic assays on WT and *Trem2*^−/−^ BMDM, given that we couldn’t obtain sufficient quantities of viable cellular material from macrophage of FACS sorting from the WT and Trem2^−/−^ mice with UUO. Then, transcriptomic data were analyzed for differentially expressed genes (DEGs) with a false discovery rate (FDR) < 0.05 and |Fold Change| > 2 (Fig. 5a). As shown in Fig. 5b, we evaluated functional associations according to the Kyoto Encyclopedia of Genes and Genomes (KEGG) pathway enrichment analyses. We noted that the apoptotic pathway was in the top 15 enrichment pathways, confirming that TREM2 deficiency influenced the regulation of apoptosis. In the cluster heatmap of differential gene expression shown in Fig. 5c, a significant downregulation in the expression of anti-apoptosis-related proteins, such as Bcl2, Bcl2l1, and Fos was observed. Additionally, the expression of mTOR signaling-related proteins, such as Slc7a5 and Igf1 was significantly downregulated. In summary, the transcriptomic data obtained from BMDM indicate that TREM2 deficiency may inhibit macrophage survival through downregulation of the mTOR pathway.

The mTOR signaling is a classical pathway that regulates gene transcription and protein synthesis to regulate cell proliferation and immune cell differentiation and is closely associated with cell survival and apoptosis [38, 39]. We then assessed the expression of key proteins involved in mTOR signaling in WT and *Trem2*^−/−^ BMDM. Immunoblotting results revealed that the phosphorylation levels of mTOR and Akt were significantly reduced in *Trem2*^−/−^ BMDM, whereas the expression level of p70S6 kinase (S6K), a downstream molecule of mTORC1, was significantly upregulated (Fig. 5d). Given the inhibitory effect of TREM2 deficiency on mTOR activation in BMDM, we attempted to overcome this deficiency by employing alternative approaches that bypassed TREM2. The creatine analog cyclocreatine produces a long-lasting phosphate upon phosphorylation, which is effective in maintaining cellular ATP levels during increased energy demand [40]. We added cyclocreatine to BMDM cultures and performed a CCK-8 assay, which revealed a significant improvement in survival of *Trem2*^−/−^ BMDM, and in some cases, the addition of cyclocreatine even led to a reversal of survival outcome (Fig. 5e), thus validating our conclusion that TREM2 regulates macrophage survival through the Akt/mTOR pathway.

### TREM2 deficiency promoted macrophage polarization in UUO kidneys and BMDM

**Fig. 6:**
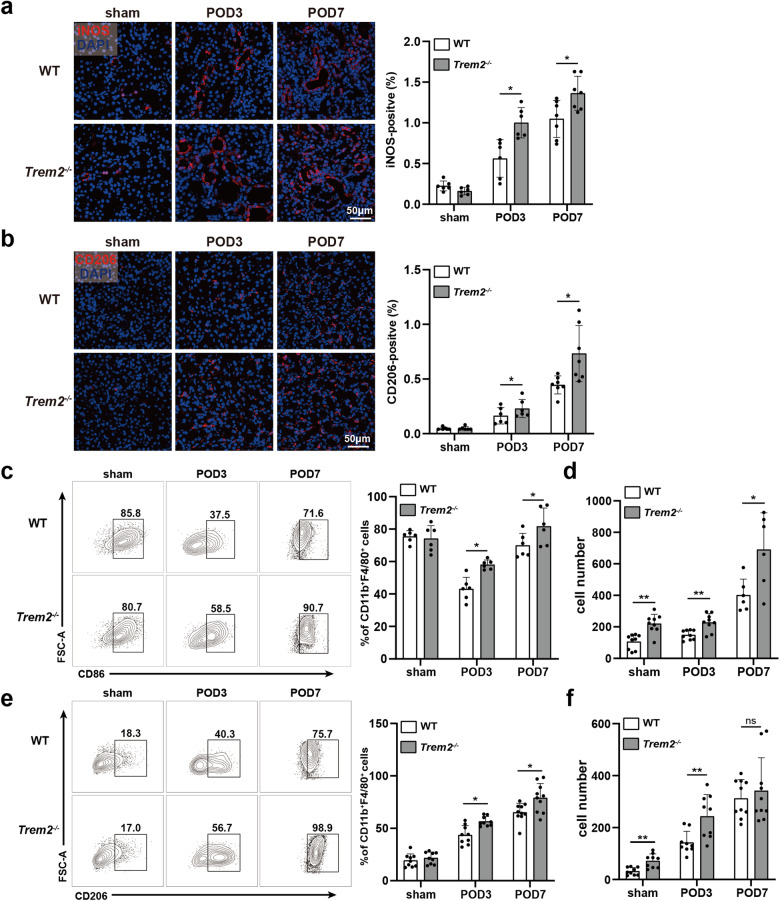
TREM2 deficiency promotes macrophage polarization. **a** Fluorescent representative images of iNOS^+^ cells in UUO kidneys of WT and *Trem2*^−/−^ mice. Quantification of the percentage of area occupied by iNOS^+^ cells in the two groups of mice was performed using Image Pro Plus 6.0 software *n* = 6–7. Red: iNOS, blue: DAPI. **b** Fluorescent representative images of CD206^+^ cells in the UUO kidneys of WT and *Trem2*^−/−^ mice. The percentage of area occupied by red fluorescence of CD206^+^ cells in both groups of mice was quantified using Image Pro Plus 6.0 software, *n* = 6–7. Red: CD206, blue: DAPI. Flow cytometry representative images of (**c**) CD86^+^ (*n* = 6) and (**e**) CD206^+^ (*n* = 9–10) macrophages in the UUO kidneys of two groups of mice (gated on CD11b^+^F4/80^+^ cells). Absolute counts of (**d**) CD86^+^ (*n* = 6–9) and (**f**) CD206^+^ (*n* = 9–10) macrophages in the two groups of mice. *P < 0.05, **P < 0.01.

In addition to cell survival, macrophage polarization has been reported to be a key factor in UUO progression [41–43]. Through IF detection of iNOS and the mannose receptor CD206 in the UUO kidney, we noted an increased proportion of both iNOS^+^ cells and CD206^+^ cells in kidneys of *Trem2*^−/−^ mice on POD3 and POD7 (Fig. 6a, b). Additionally, both F4/80-positive iNOS^+^ and Arg1^+^ cells were elevated in the kidney tissues of POD7 mice (Fig. S4a, b). Subsequently, we assessed the percentages of M1 and M2 macrophages in the UUO kidneys using flow cytometry. The results revealed a significant increase in the proportion of CD86^+^ macrophages (M1) in the UUO kidneys of *Trem2*^−/−^ mice on POD3 and POD7 (Fig. 6c). Moreover, M1 macrophages are proinflammatory cells characterized by high expression of MHC II, CD80, CD86, CD38, and TLR4 [44]. MHC II^+^ macrophages were also significantly elevated in the UUO kidneys of *Trem2*^−/−^ mice on POD3 (Fig. S4c, d). Interestingly, TREM2 deficiency resulted in a significant increase in M1 macrophages in the kidneys of sham mice (Figs. 6d and S4e). This suggests that TREM2 exerts a regulatory effect on macrophage polarization in kidneys that are not obstructed. Furthermore, the proportion of CD206^+^ cells was significantly higher in the UUO kidneys of *Trem2*^−/−^ mice (Fig. 6e). In addition, absolute counts of CD206^+^ cells indicated that TREM2 deficiency significantly increased M2 macrophages in UUO kidneys (Fig. 6f). These data suggest that TREM2 deficiency significantly promoted macrophage polarization toward M2 in UUO kidneys.

**Fig. 7:**
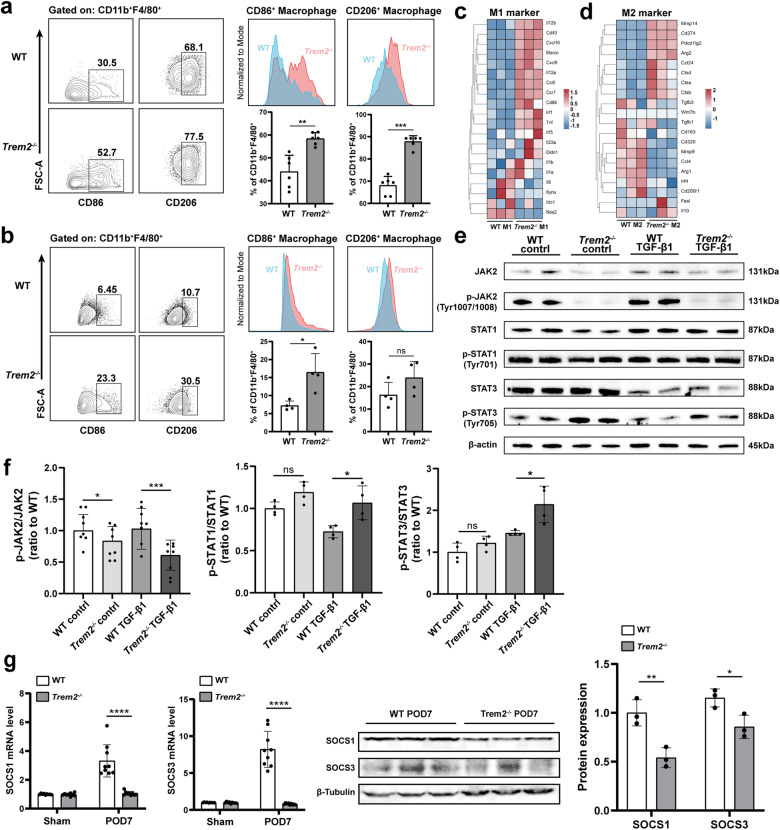
TREM2 deficiency promotes BMDM polarization via the JAK2-STAT1/3 pathway. **a** Flow representative images of BMDM polarized to M1 and M2 following LPS or IL-4 stimulation (gated on CD11b^+^F4/80^+^ cells), superimposed images of two sets of peak and statistical analysis of BMDM polarization, *n* = 6. **b** Flow representative images of BMDM polarized to M1 and M2 following TGF-β1 stimulation (gated on CD11b^+^F4/80^+^ cells) respectively, superimposed images of two sets of peak and statistical analysis of BMDM polarization, *n* = 4. Heatmap of mRNA levels of BMDM (**c**) M1- and (**d**) M2-related markers. **e** Protein expression levels of JAK2, p-JAK2 (Tyr1007/1008), STAT1, p-STAT1 (Tyr701), STAT3, and p-STAT3 (Tyr705) were detected by western blotting in WT and *Trem2*^−/−^ BMDM in control and TGF-β1 groups, respectively. **f** Western blotting of the key proteins associated with the JAK-STAT pathway was analyzed in the grayscale, *n* = 4–8. **g** The measurement of mRNA and protein levels of SOCS1 and SOCS3, *n* = 3. *P < 0.05, **P < 0.01, ***P < 0.001, ****P < 0.0001.

Consistent with our in vivo findings, the proportion of *Trem2*^−/−^ BMDM polarized into M1 cells following LPS stimulation was higher compared to WT BMDM. Moreover, the proportion of *Trem2*^−/−^ BMDM polarized into M2 was notably higher than that of WT BMDM following IL-4 stimulation (Fig. 7a). To reflect the UUO environment as far as possible in vitro, we drew on insights from previous studies [45, 46] and used TGF-β1 to stimulate the BMDM. The TGF-β superfamily is the most widely studied macrophage-derived growth factor and is a key inducer of the epithelial-mesenchymal transition (EMT) process in renal fibrosis [47]. Our data reveal that following TGF-β1 stimulation, expression of the M1 marker CD86 by *Trem2*^−/−^ BMDM was significantly higher, while expression of the M2 marker CD206 tended to increase, although not significantly (Fig. 7b). Furthermore, we conducted transcriptomic sequencing analysis on the M0, M1, and M2 subtypes of BMDM, respectively. Our findings reveal a general upregulation of key markers associated with M1 and M2 polarization in *Trem2*^−/−^ BMDM (Fig. 7c, d), which further suggests that TREM2 deficiency promotes polarization of macrophages towards M1 and M2 phenotypes.

### TREM2 deficiency regulated JAK-STAT signaling pathway

The Janus kinase (JAK)-signal transducer and activator of transcription (STAT) signaling pathway has been studied extensively and found to play a crucial role in kidney inflammation by regulating the expression of diverse inflammatory molecules [48, 49], which in turn regulate M1/M2 polarization of macrophages [50]. To verify whether TREM2 exerted a regulatory effect on macrophage polarization through the JAK-STAT pathway, we assessed the expression of key proteins of JAK-STAT signaling in WT and *Trem2*^−/−^ BMDM in control and TGF-β1-stimulated groups, respectively. The results show that TREM2 deficiency substantially reduced phosphorylation levels of JAK2 in both control and TGF-β1-stimulated groups. Additionally, the phosphorylation levels of STAT1 and STAT3 were significantly increased in *Trem2*^−/−^ BMDM following TGF-β1 stimulation (Fig. 7e, f). In summary, our findings suggest that TREM2 deficiency promotes macrophage polarization by regulating JAK2-STAT1/3 signaling, which in turn, exacerbates UUO tubular injury, renal inflammation, and interstitial fibrosis.

Moreover, a recent study has reported that crosstalk exists between mTOR and STAT signaling cascades through the suppressor of cytokine signaling (SOCS) signaling [51]. Additionally, the SOCS family of proteins are negative feedback inhibitors of signaling induced by cytokines that act via the JAK/STAT pathway [52]. SOCS1 expression impedes STAT5 and STAT3 protein phosphorylation [53]. LPS pretreatment-induced SOCS3 inhibits IFN-β-induced STAT1 phosphorylation [54]. Therefore, the expression of the SOCS1 and SOCS3 was then evaluated. As shown in Fig. 7g, the mRNA and protein levels of SOCS1 and SOCS3 were significantly reduced, suggesting that mTOR may regulate both SOCS1/STAT3 and SOCS3/STAT1 signaling, which ultimately affected macrophage M1/M2 polarization.

### TREM2 deficiency aggravated IRI-induced renal injury and fibrosis and promoted macrophage polarization in mice

**Fig. 8:**
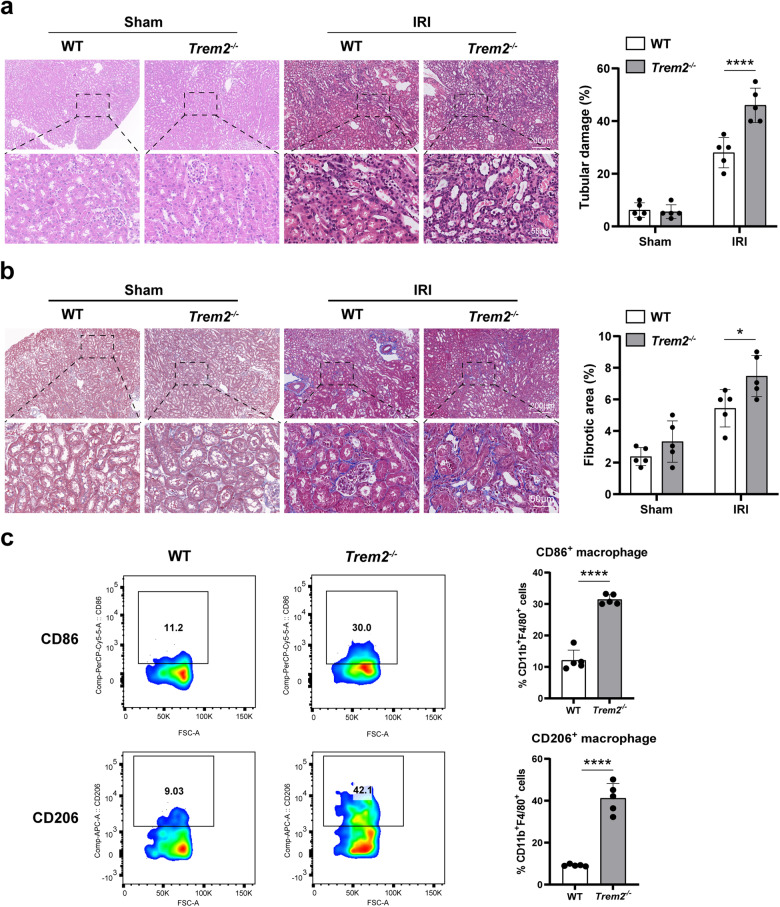
TREM2 deficiency aggravated IRI-induced renal injury and fibrosis and promoted macrophage polarization in mice. **a** Left; H&E staining of IRI kidneys from WT and *Trem2*^−/−^ mice. right; H&E results were assessed and quantified as the percentage of the tubular damage area. **b** Masson staining of IRI kidney tissues from WT and *Trem2*^−/−^ mice; results were quantified by Image Pro Plus 6.0 software to assess the level of renal fibrosis in mice. **c** Flow cytometry representative images of CD86^+^ and CD206^+^ macrophages in the IRI kidneys of two groups of mice (gated on CD11b^+^F4/80^+^ cells). *n* = 5 per group. *P < 0.05, ****P < 0.0001.

Given that, in addition to UUO, the IRI procedure is also a well-recognized experimental model, which is widely used to study renal tubular injury and fibrosis [22, 23]. Therefore, we next conducted an IRI mice model to further confirm the effect of TREM2 on kidney injury and macrophage polarization. As expected, the H&E staining displayed that TREM2 deficiency remarkably aggravated renal injury induced by IRI on POD7 (Fig. 8a). Meanwhile, the fibrosis levels of mice kidney tissues were significantly increased after TREM2 knockout (Fig. 8b). Furthermore, consistent with the UUO results, the M1 macrophages (CD86^+^) and M2 macrophages (CD206^+^) both exhibited significant upregulation in the mice kidney according to flow cytometry data (Fig. 8c). From the above data, TREM2 deficiency aggravated IRI-induced renal injury and fibrosis and promoted macrophage polarization in mice.

## Discussion

In the present study, we found that TREM2 expression was induced in the kidneys of UUO mice, consistent with observations in models of cerebral hemorrhage [55] and Alzheimer’s disease (AD) [56]. Accordingly, we hypothesized that TREM2 expression in UUO kidneys might be associated with the modulation of renal injury. To investigate the role of TREM2 in UUO-mediated kidney injury, we used *Trem2*^−/−^ mice and validated the predominant expression of TREM2 in macrophages of UUO kidneys. Moreover, our IF findings indicated that, in addition to its expression on macrophage membranes, TREM2 was also evident in intercellular spaces, indicating the presence of secreted/shed soluble(s) TREM2 within the kidney. Thus, sTREM2 may play a crucial role in the modulation of the pathobiology of the UUO process as both membrane-associated and soluble protein forms, which may become the direction of our further research. Notably, in addition to macrophage, TREM2 is also expressed on part of dendritic cells. A previous study [57] has reported that TREM2-mediated dendritic cell-induced NO suppresses Th17 activation in UUO mice. Therefore, further investigation is necessary to explore the effect of TREM2 on the phenotype and function of dendritic cells. Furthermore, they found that tubular damage, myofibroblast accumulation, and fibrosis were not different between WT and *Trem2*^−/−^ mice on day 7 of the UUO model, but significant differences became apparent on day 14. Possible reasons for the differences in the results of our study may be differences in the details of the modeling process, such as the degree of obstruction, the duration of surgery, and the surgical environment. To ensure the reliability of our results, we then utilized another well-recognized experimental model, the IRI model, which is more widely used to study renal tubular injury and fibrosis. As expected, we got a consistent conclusion. On POD7 following IRI, the fibrosis level was significantly promoted in *Trem2*^−/−^ mice.

The TREM2 innate immune receptor has been extensively reported to promote macrophage survival under stress conditions [58]. Our data show that TREM2 deficiency reduced macrophage infiltration in mouse UUO kidneys. Correspondingly, our in vitro studies revealed that TREM2 deficiency inhibited macrophage survival and promoted apoptosis. Moreover, transcriptomic sequencing data of BMDM corroborated our findings. These observations align with previous studies that have demonstrated the ability of TREM2 to promote the survival of microglia, the primary innate immune cells of the CNS, that are similar to macrophages, and their proliferation in models of neurodegenerative diseases, such as AD and multiple sclerosis [59, 60].

Previous studies have documented that the absence of mTOR signaling in TREM2-deficient microglia is associated with compensatory upregulation of autophagy in patients with AD, both in vivo and in vitro [58]. Contrary to the inflammatory scenario we observed in UUO kidneys, microglial expression of inflammatory mediators is reported to be weaker in 5XFAD transgenic (AD model) mice lacking TREM2 [61]. However, we did not find any changes in autophagy (data not shown). Similarly, defective mTOR activation in TREM2-deficient macrophages resulted in macrophage dysfunction, reduced cell viability, and proliferation, as confirmed by an increase in caspase 3 activation and an increased incidence of apoptosis in *Trem2*^−/−^ BMDM in vitro. Thus, while mTOR activation deficiency in TREM2-deficient macrophages may have reduced macrophage infiltration in UUO kidneys, inflammation in the kidney instead increased. This suggests that TREM2 may regulate mTOR signaling and influence events triggered by UUO via other pathways.

Multiple macrophage subtypes that release numerous cytokines play key roles in pathophysiological processes. Thus, M1 and M2 phenotypic polarization of macrophages may directly influence UUO pathologic events. While TREM2 has been reported to regulate macrophage polarization in several studies [62, 63], our findings reveal for the first time that TREM2 deficiency enhances macrophage activation and polarization toward both M1 and M2 cells in UUO kidneys. Among them, M1 macrophage markers, including iNOS, CD86, and MHC II, were significantly elevated, which explains part of the reason why TREM2 deficiency promotes renal injury and macrophage apoptosis. On the other hand, macrophage polarization towards M2 was consistent with the findings of Yang et al. showing that TREM2 activation in the CNS inhibits microglia/macrophage activation [55] and increases the number of CD206^+^ immunosuppressive macrophages [64]. However, it is a bit contradictory to our previous results because M2 is usually considered to have anti-inflammatory effects, contrary to M1. This may be due to the strong plasticity of macrophages, which differ significantly in different tissues, especially microglia in brain injury. This specificity of microglia may be due to their unique origin, resulting in a different transcriptome and epigenome from macrophages in other tissues [65]. This deserves more extensive research and study. Furthermore, Lisi et al. identified that proinflammatory activation of glioma cells induced a shift in microglia from a predominantly M2b phenotype to a mixture of M1 and M2a/b polarized cells [66]. Likewise, in our study, TREM2 deficiency promoted the expression of CD86, CD206, MHC II, iNOS, and Arg1 in macrophages, which suggested that macrophages were induced to shift toward a mixture of M1 and M2a/b by TREM2 deficiency. In sum, a spectrum or mixed phenotype of macrophage polarization may be present in TREM2 deficiency. Beyond the role of TREM2 in the mixed phenotype of macrophage polarization, how TREM2 regulates the efferocytosis of macrophage deserves further research. Lastly, TREM2 is expressed in the myeloid cells besides macrophages, which means conditional KO including Lyz2-Cre or CXCR3-Cre and TREM2 floxed mice would be more suitable to investigate their mechanisms in-depth.

Numerous studies have reported that macrophage phenotype and activation are regulated by JAK-STAT signaling [67, 68]. To investigate whether TREM2 might regulate macrophage polarization through JAK-STAT signaling, we assessed differences in expression levels of JAK2, STAT1, and STAT3, key proteins of the JAK-STAT pathway. Our data confirmed that TREM2 deficiency promoted macrophage polarization toward the M1 phenotype by upregulating STAT1 activation, and promoted macrophage polarization toward the M2 phenotype by upregulating STAT3 activation.

Our study revealed that TREM2 is a promising therapeutic target for CKD. Therefore, specific small molecules that activate TREM2 could be potentially developed to treat CKD in the future.

## Conclusion

The present findings suggest that additional mechanisms whereby the protective effects of macrophage-expressed and secreted TREM2 on UUO kidneys are mediated merit investigation. In conclusion, our findings indicate that TREM2 deficiency inhibits macrophage survival and promotes apoptosis through the downregulation of Akt/mTOR signaling and that macrophage polarization via the JAK2-STAT1/3 pathways may exacerbate tubular injury, renal apoptosis, inflammation, and fibrosis in mice.

### Supplementary information

Supplement material

Original western blots

## Data Availability

Complete and uncropped Western blotting imprints are uploaded as Supplementary Material. The raw sequencing data from this study have been deposited in the Genome Sequence Archive in BIG Data Center (http://bigd.big/ac.cn/), Beijing Institute of Genomics (BIG), Chinese Academy of Sciences, under the accession number: GRA0117711.
