# Supplementary material for: TREM2 deficiency aggravates renal injury by promoting macrophage apoptosis and polarization via the JAK-STAT pathway in mice
Source: Cell Death Dis. 2024 Jun 7;15(6):401. doi: 10.1038/s41419-024-06756-w (PMC11161629; doi:10.1038/s41419-024-06756-w)
Supplement: Supplementary file 1 — Supplement material [file 41419_2024_6756_MOESM1_ESM.docx]

**Supplementary Material**

**Supplement Table S1 | Primers for real time-PCR**

| Gene | Forward 5’-3’ | | Reverse 5’-3’ |
| --- | --- | --- | --- |
| Trem2 | CTGCTGGCAAAGGAAAGGTG | TTCCTGGAGGTGCTGTGTTC | |
| Kim-1 | CGTGGCTATCACCAGGTACATACT | GGTTCTGCAAAGCTTCAATC | |
| Tnf-α | CCTCACACTCAGATCATCTTC | CGGCTGGCACCACTAGTTG | |
| Il-6 | CTTGGGACTGATGCTGGTGACA | GCCTCCGACTTGTGAAGTGGTA | |
| Il-1β | TCGCAGCAGCACATCAACAAGA | CCACGGGAAAGACACAGGTAGC | |
| SOCS1 | CCCTCTTAACCCGGTACTCC | CTCCCACGTGGTTCCAGAAA | |
| SOCS3 | GCAGGAGAGCGGATTCTACT | TGGATGCGTAGGTTCTTGGT | |
| actin | CATCCGTAAAGACCTCTATGCCAAC | ATGGAGCCACCGATCCACA | |

**Supplement Table S2 | Summary of antibodies used**

| Antibodies | Supplier | Identifier |
| --- | --- | --- |
| Anti-mouse caspase3/p17/p19 | Proteintech, Rosemont, IL, USA | 19677-1-AP |
| Bcl-2 (D17C4) | Cell Signaling Technology, Danvers, MA, USA | #3498 |
| Akt | Cell Signaling Technology, Danvers, MA, USA | #9272 |
| Phosphor-Akt (Sre473) | Cell Signaling Technology, Danvers, MA, USA | #9271 |
| mTOR (7C10) | Cell Signaling Technology, Danvers, MA, USA | #2983 |
| Phospho-mTOR (ser2448) | Cell Signaling Technology, Danvers, MA, USA | #5536 |
| p70 S6 Kinase (E8K6T) | Cell Signaling Technology, Danvers, MA, USA | #34475 |
| JAK2 | Zen-BioScience, Chengdu, China | #R24775 |
| Phospho-JAK2 (Tyr1007/1008) | Zen-BioScience, Chengdu, China | #R381556 |
| STAT1 | Zen-BioScience, Chengdu, China | #R25799 |
| Phospho-STAT1 (Tyr701) | Zen-BioScience, Chengdu, China | #340797 |
| STAT3 | Zen-BioScience, Chengdu, China | #251611 |
| Phospho-STAT3 (Tyr705) | Zen-BioScience, Chengdu, China | #381552 |
| SOCS1 | ImmunoWay, Plano, TX, USA | YT4362 |
| SOCS3 | ImmunoWay, Plano, TX, USA | YT5916 |
| β-Tubulin | Proteintech, Rosemont, IL, USA | 10068-1-AP |


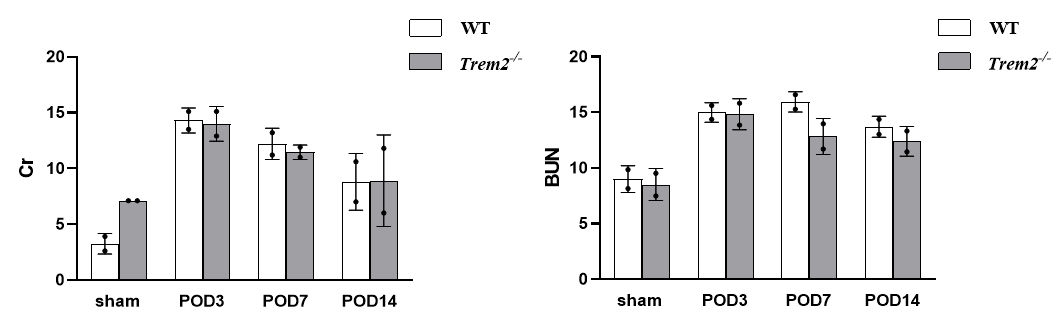


**Supplement Figure S1**

**Serum Cr and BUN levels in WT and *Trem2*^-/-^ mice.** Serum Cr and BUN levels were measured by a fully automated biochemical analyzer in both groups of mice, n=2 per group. Student *t*-test was performed. Results are presented as means ± SD. POD, postoperative day; Cr, creatinine; BUN, blood urea nitrogen.

**
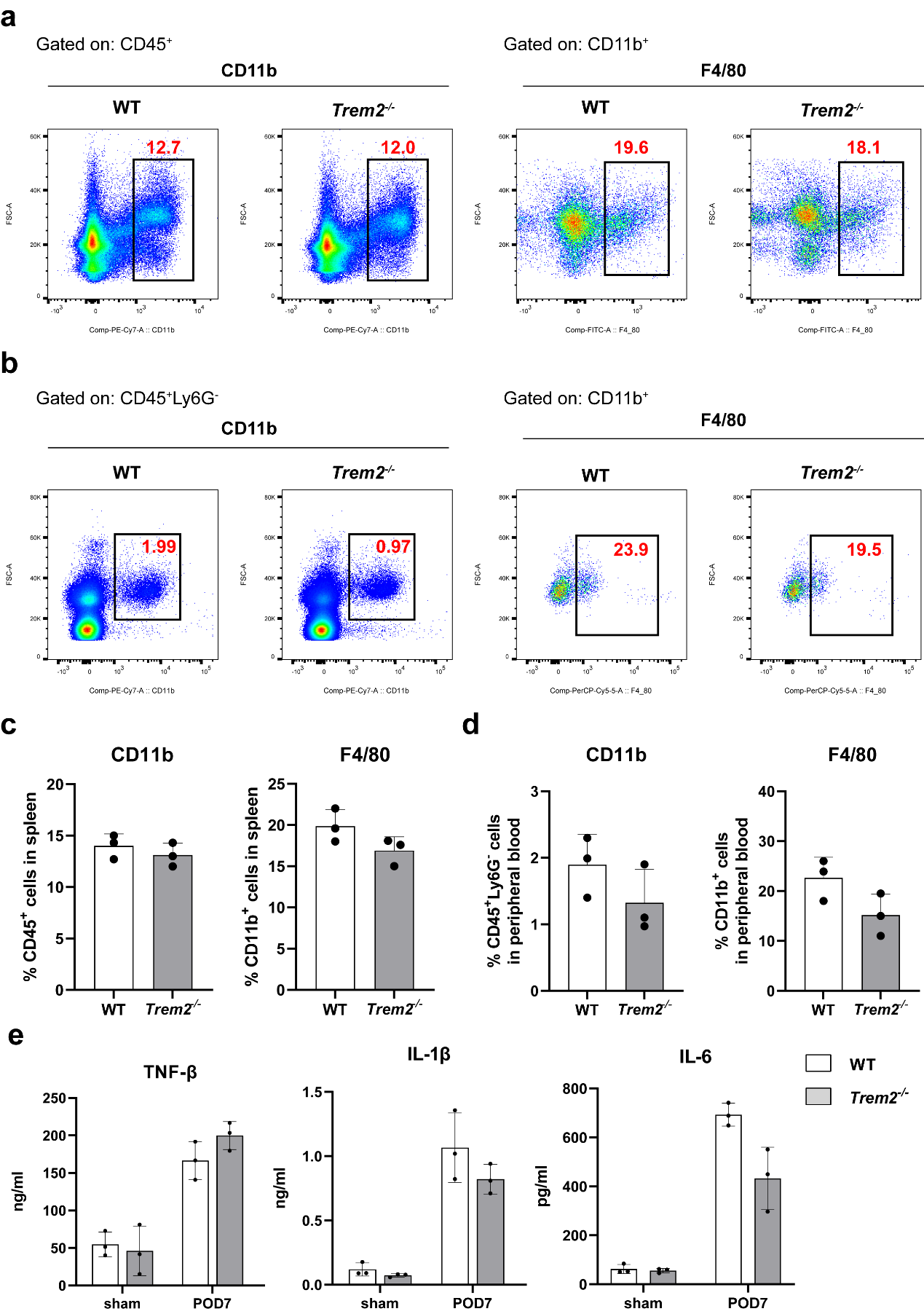
**

**Supplement Figure S2**

**The effect of TREM2 deficiency on the systemic inflammatory response.** (a) Flow cytometry representative images of CD11b^+^ and F4/80^+^ cells in the spleen tissues of UUO mice. (b) Flow cytometry representative images of CD11b^+^ and F4/80^+^ cells in the peripheral blood of UUO mice. (c) Proportion of CD11b^+^ and F4/80^+^ cells in two groups of mice. (d) Proportion of CD11b^+^ and F4/80^+^ cells in two groups of mice. (e) The levels of TNF-β, IL-1β, and IL-6 in the serum of UUO mice. n=3 per group. Student’s “*t”*-test was performed. Results are presented as means ± SD. POD, postoperative day.


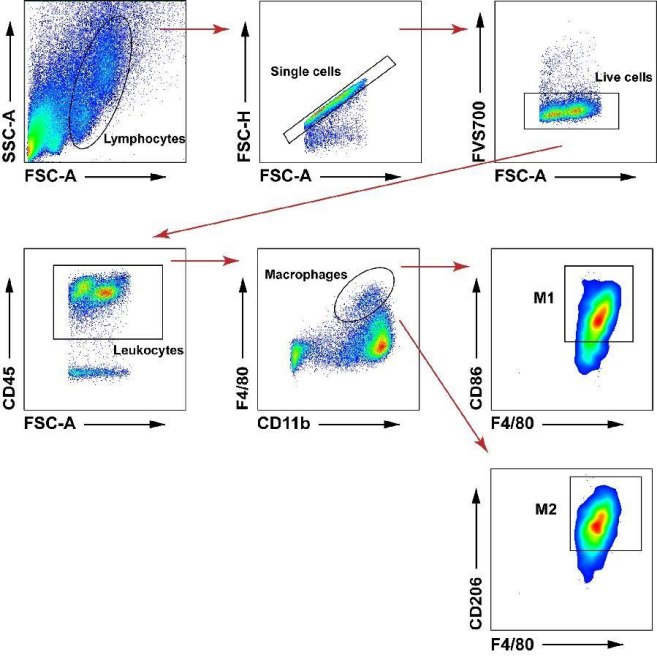


**Supplement Figure S3**

**Gating strategy for flow cytometric analysis of the kidney.** Related to Figure 4. Macrophages were identified according to the displayed gating strategy. FSC, forward scatter; SSC, side scatter; A, area; H, height.


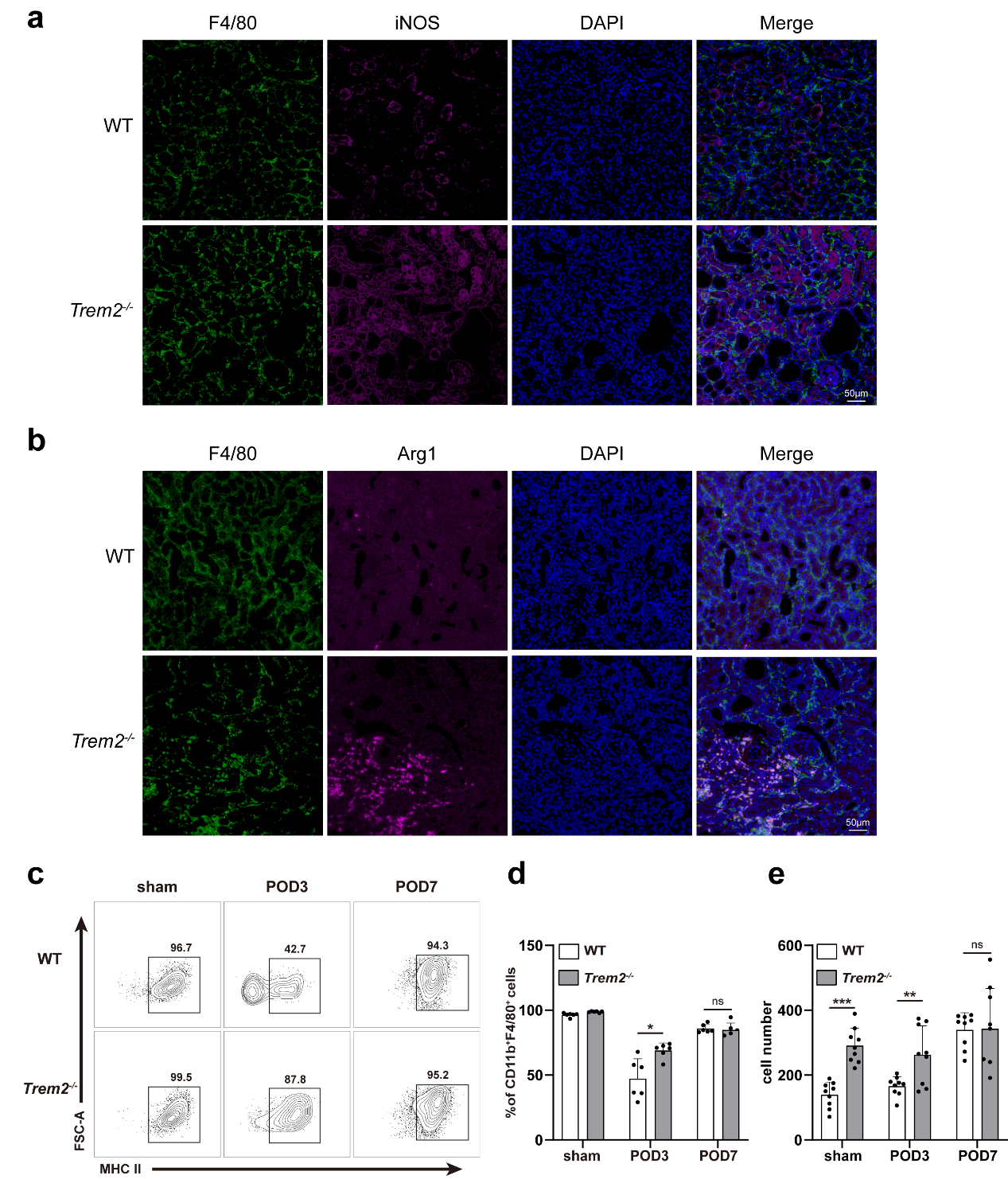


**Supplement Figure S4**

**TREM2 deficiency promotes macrophage polarization towards M1 and M2 phenotype.** (a) IF assay of F4/80 and iNOS in kidney tissues. (b) IF assay of F4/80 and Arg1 in kidney tissues. (c) Flow cytometry representative images of MHC II^+^ macrophages in the UUO kidneys of two groups of mice (gated on CD11b^+^F4/80^+^ cells). (d) Proportion and (e) absolute counts of MHC II^+^ macrophages in two groups of mice. n=6–9 per group. Student’s “*t”*-test was performed. Results are presented as means ± SD. ^*^*P* < 0.05, ^**^*P* < 0.01, ^***^*P* < 0.001. POD, postoperative day.
