# Supplementary material for: TREM2 deficiency aggravates renal injury by promoting macrophage apoptosis and polarization via the JAK-STAT pathway in mice
Source: Cell Death Dis. 2024 Jun 7;15(6):401. doi: 10.1038/s41419-024-06756-w (PMC11161629; doi:10.1038/s41419-024-06756-w)

Figure 4e western blots

caspase3

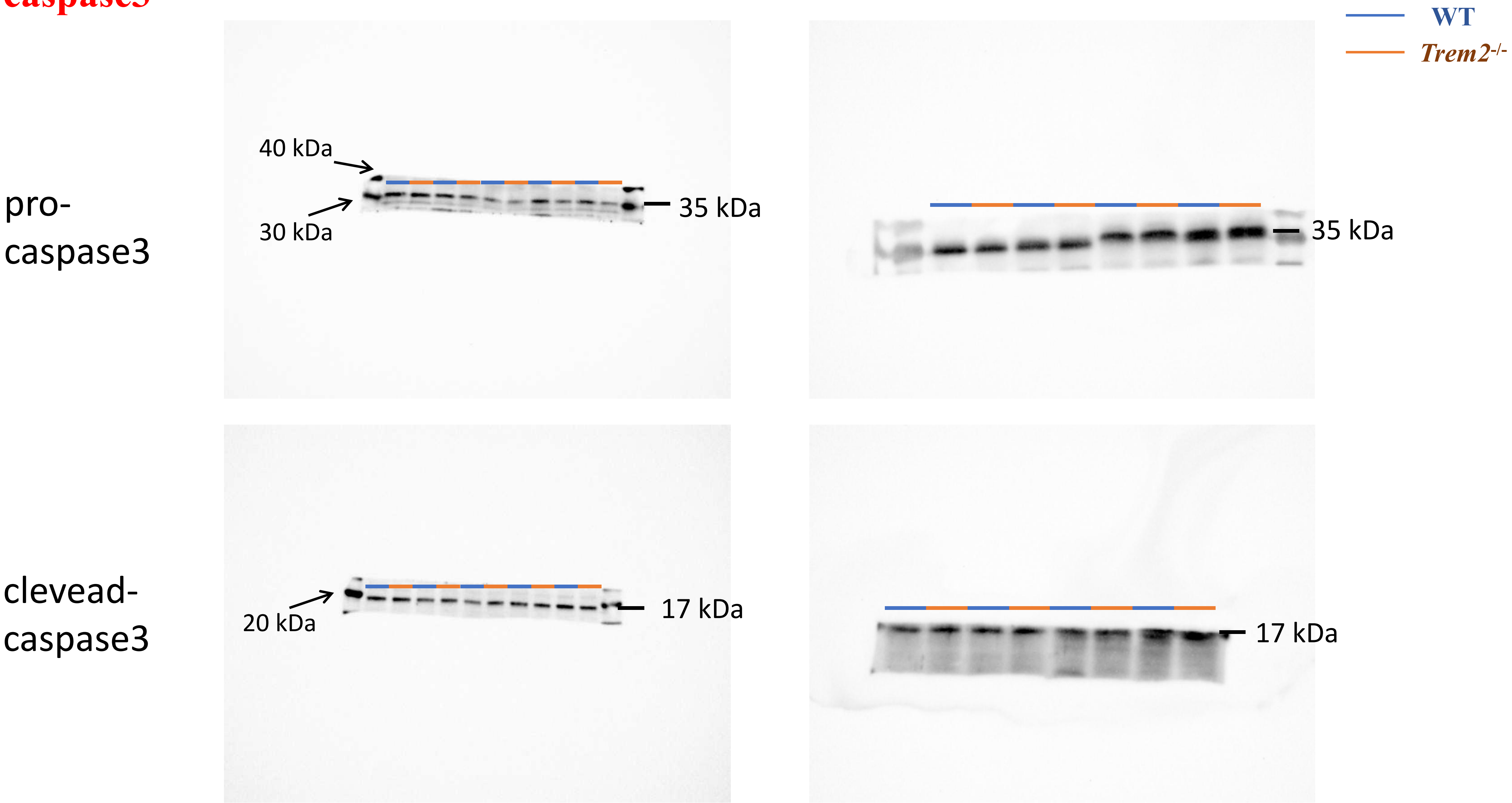

Figure 4e western blots

β-actin

WT

Trem2$^{-/-}$

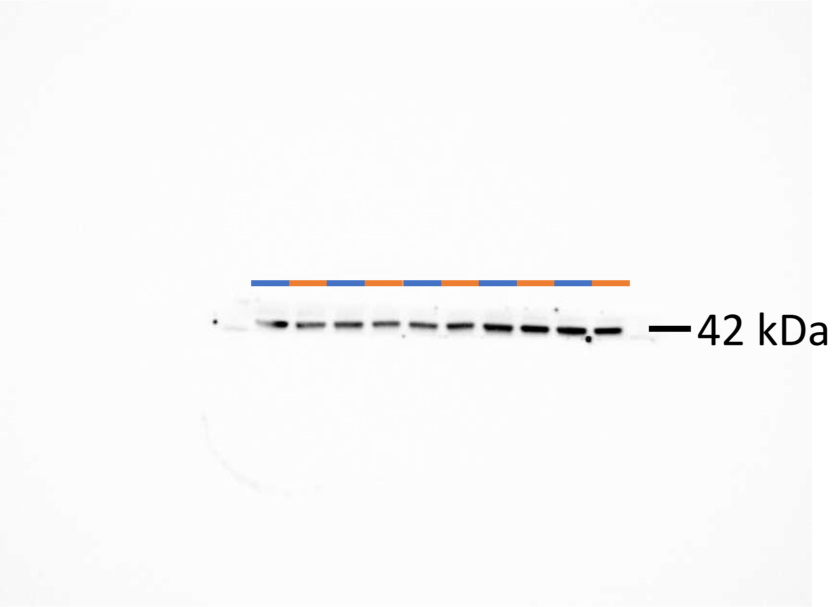

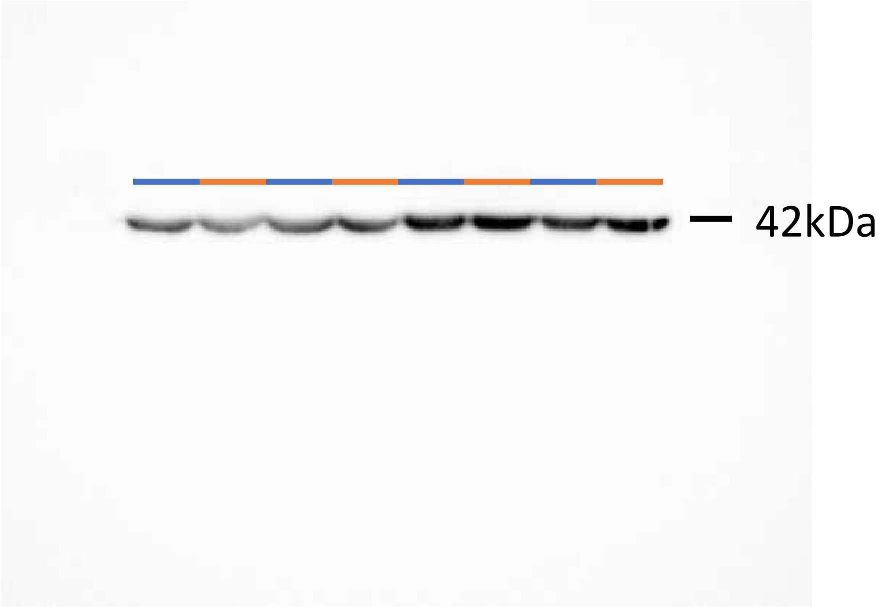

Figure 4e western blots

Bcl2

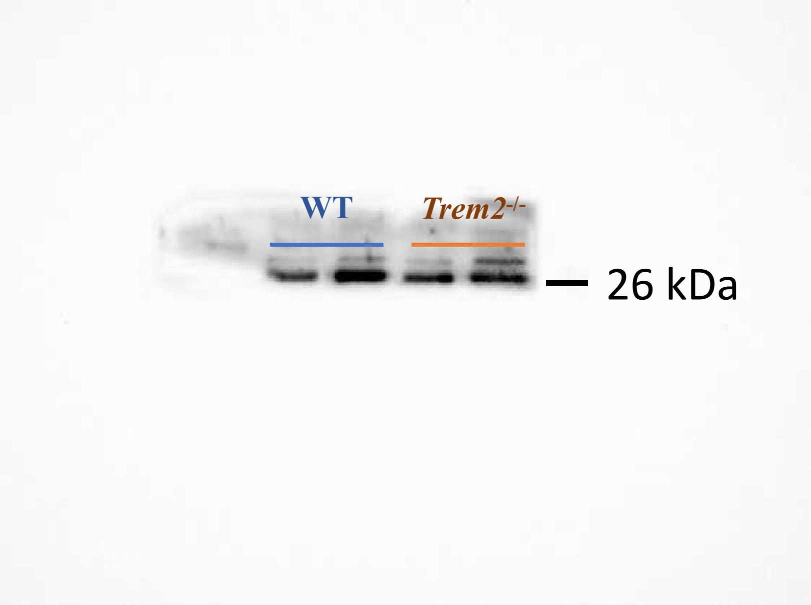

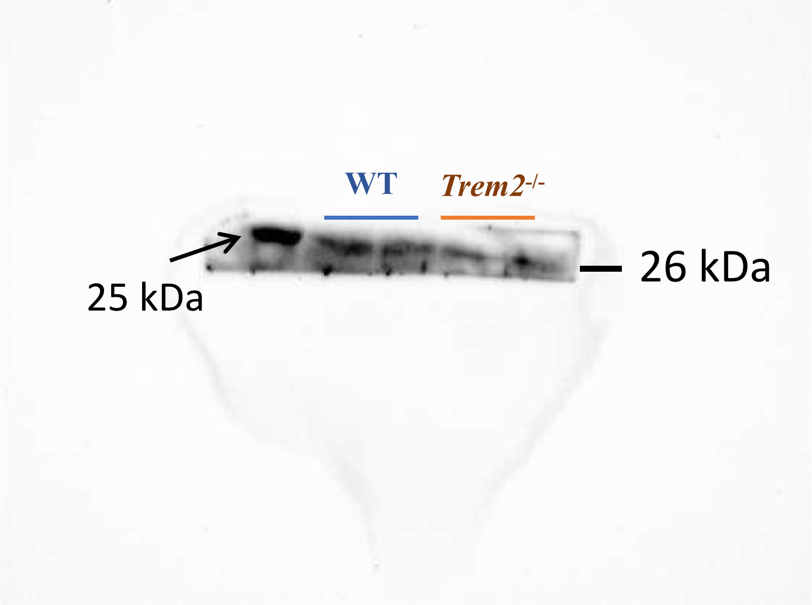

β-actin

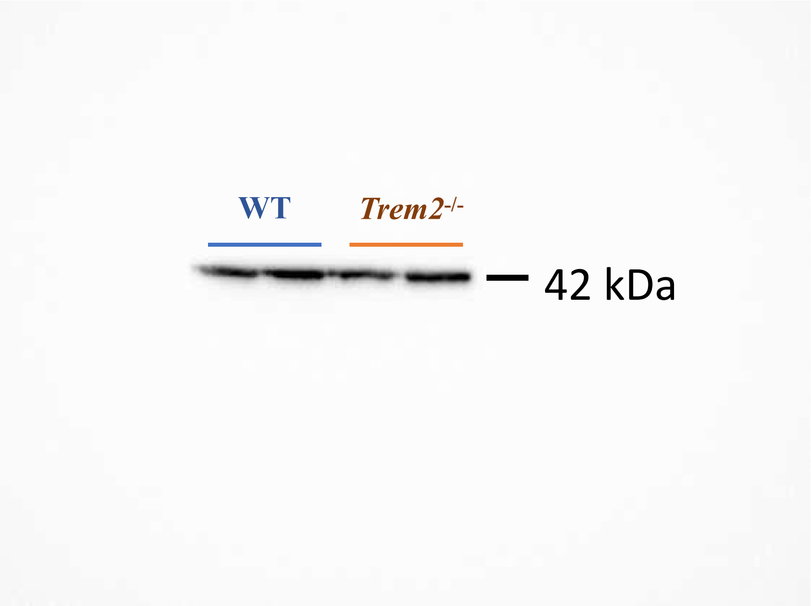

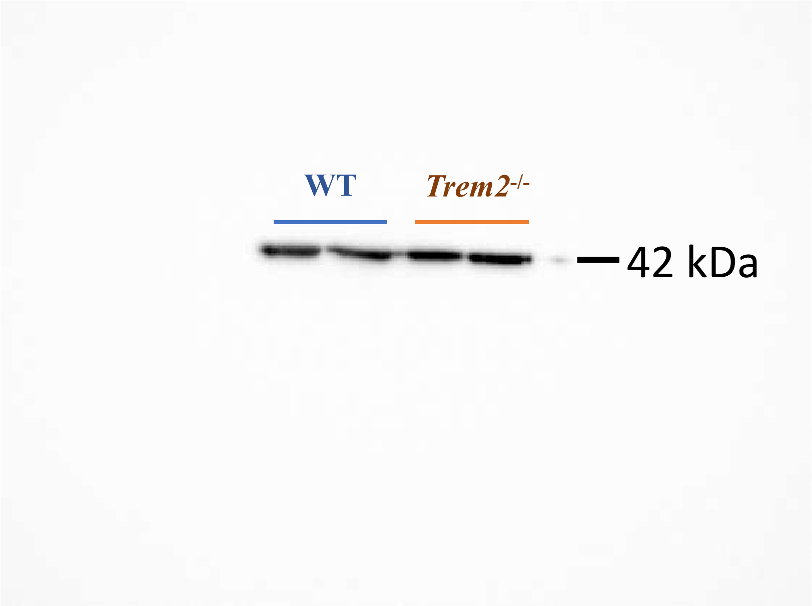

Figure 5d western blots

mTOR

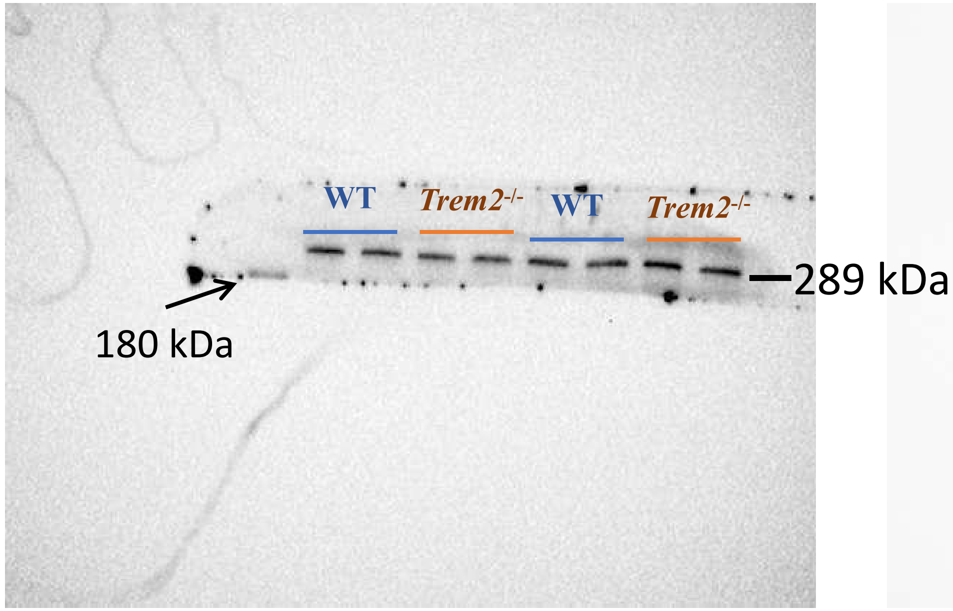

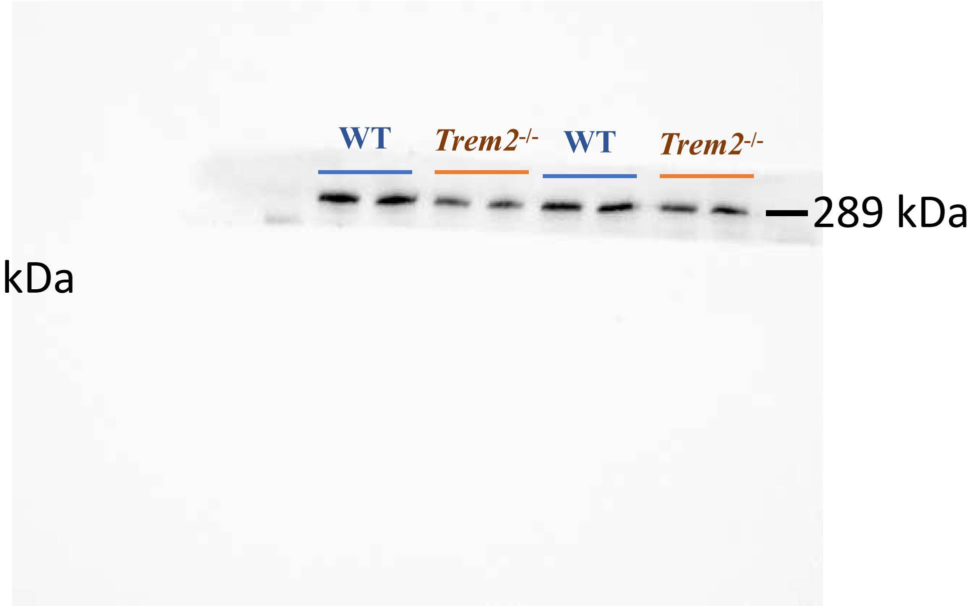

p-mTOR

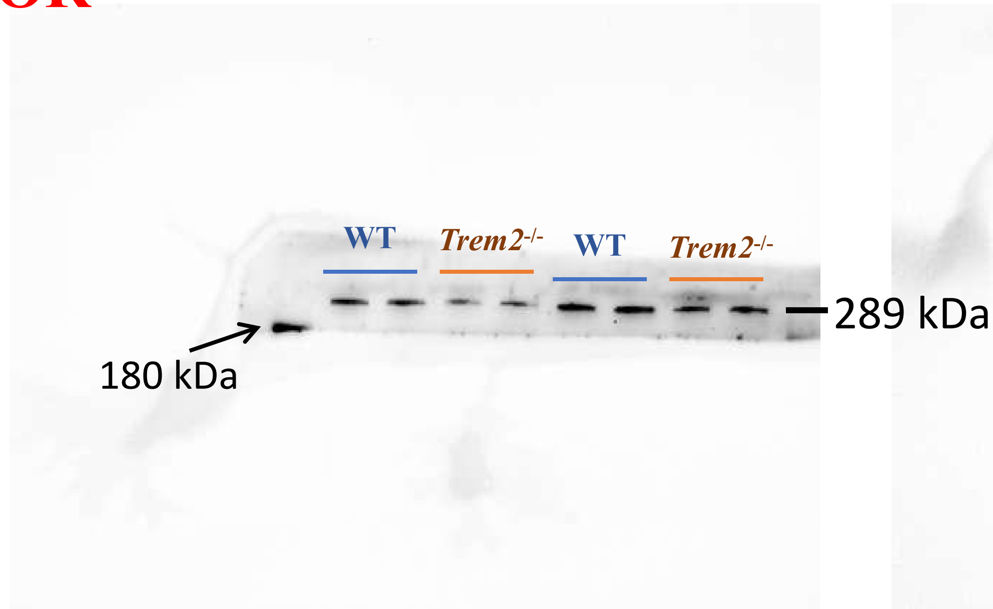

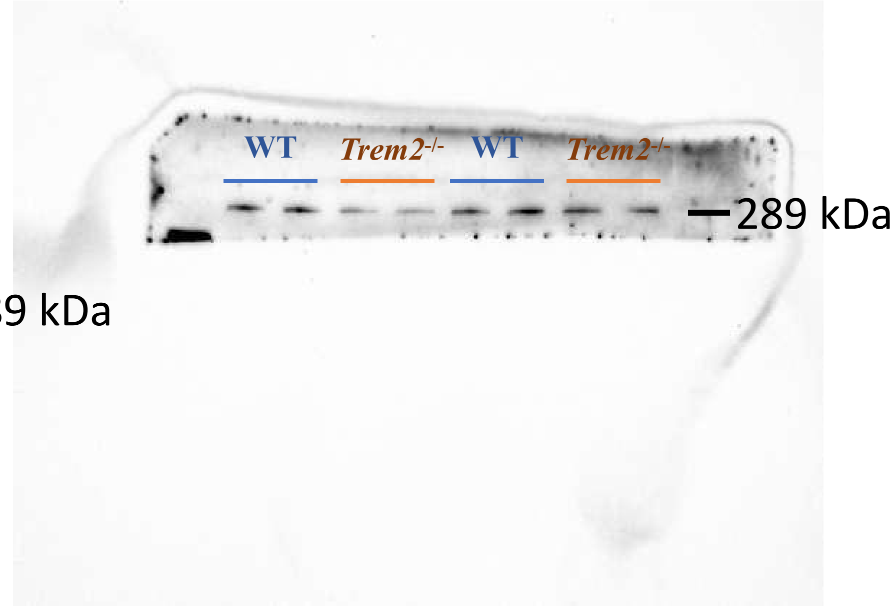

Figure 5d western blots

Akt

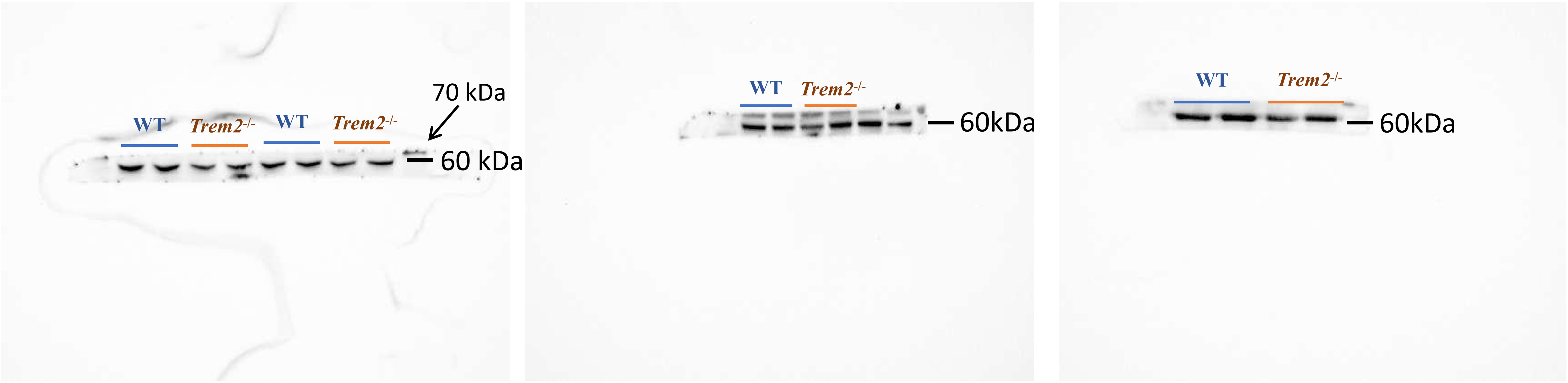

p-Akt

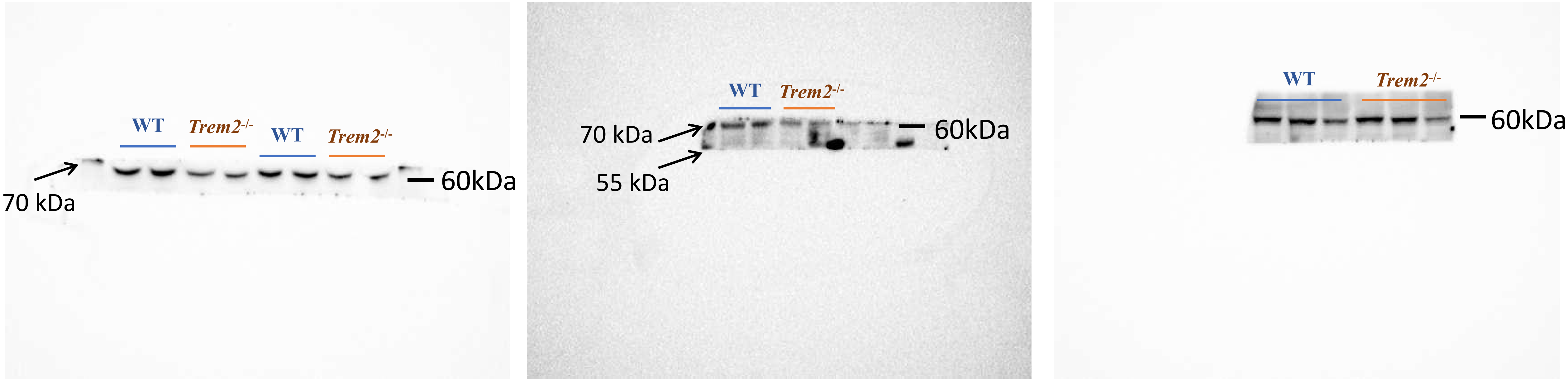

Figure 5d western blots

p70S6K

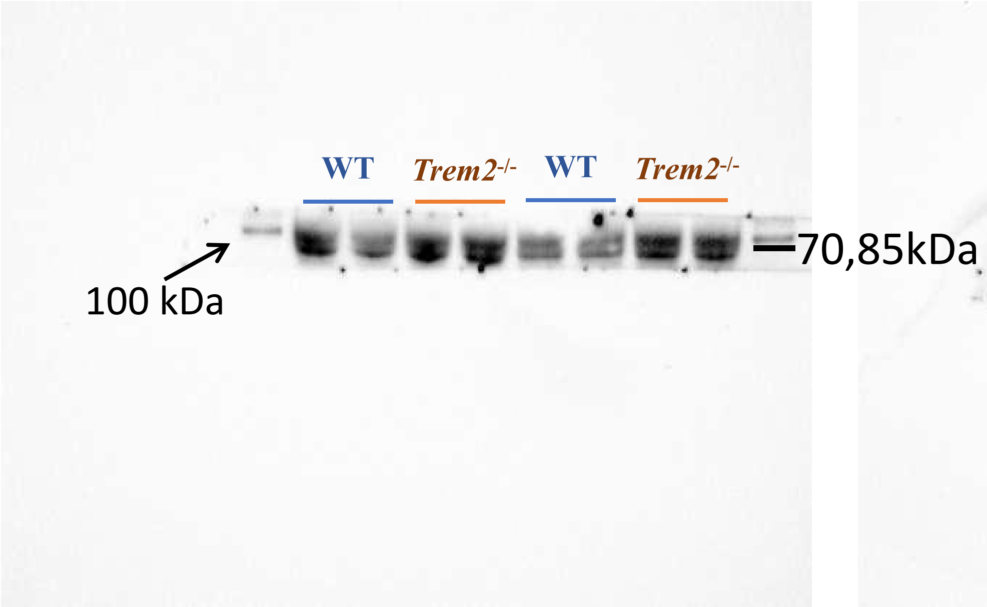

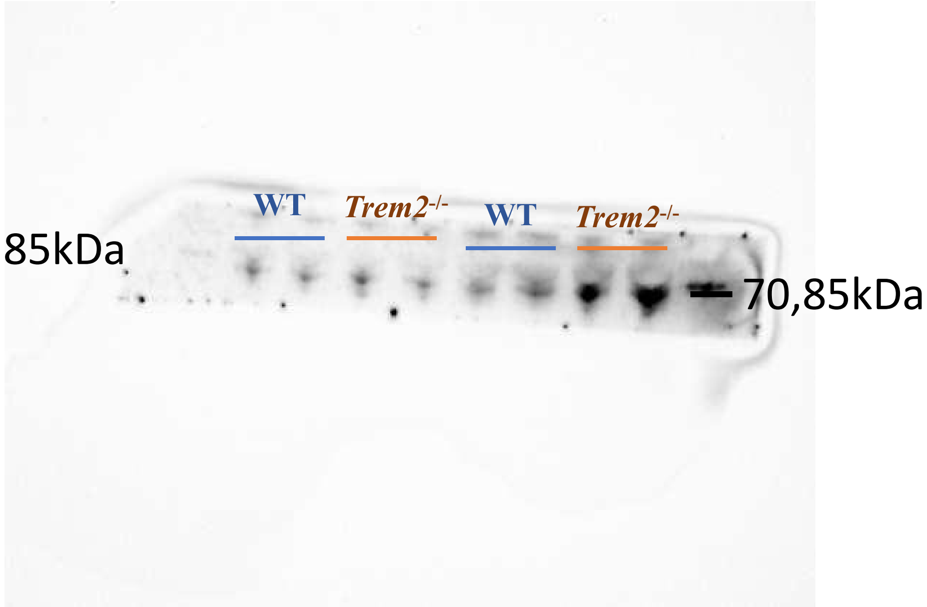

Figure 5d western blots

β-actin

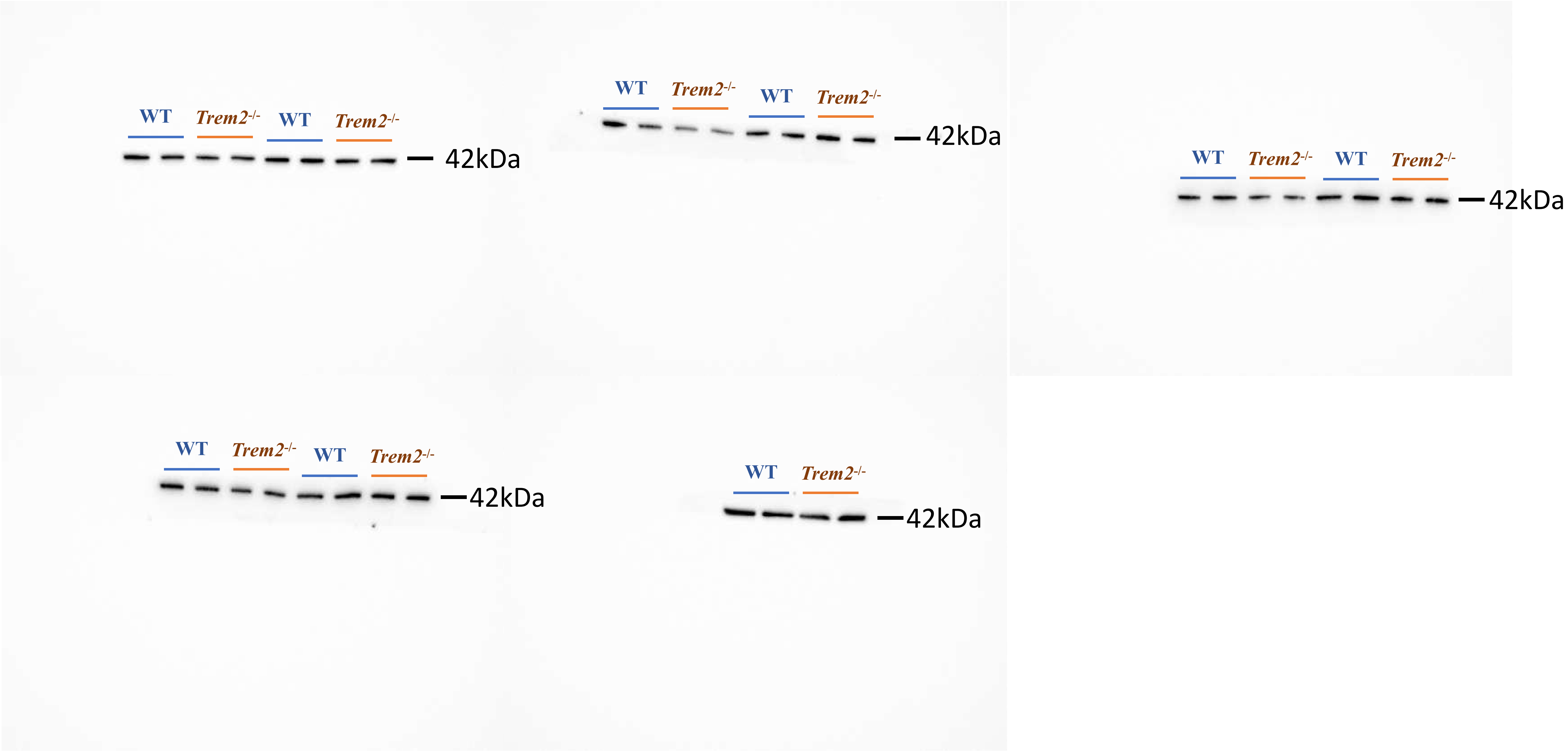

Figure 5d western blots

GAPDH

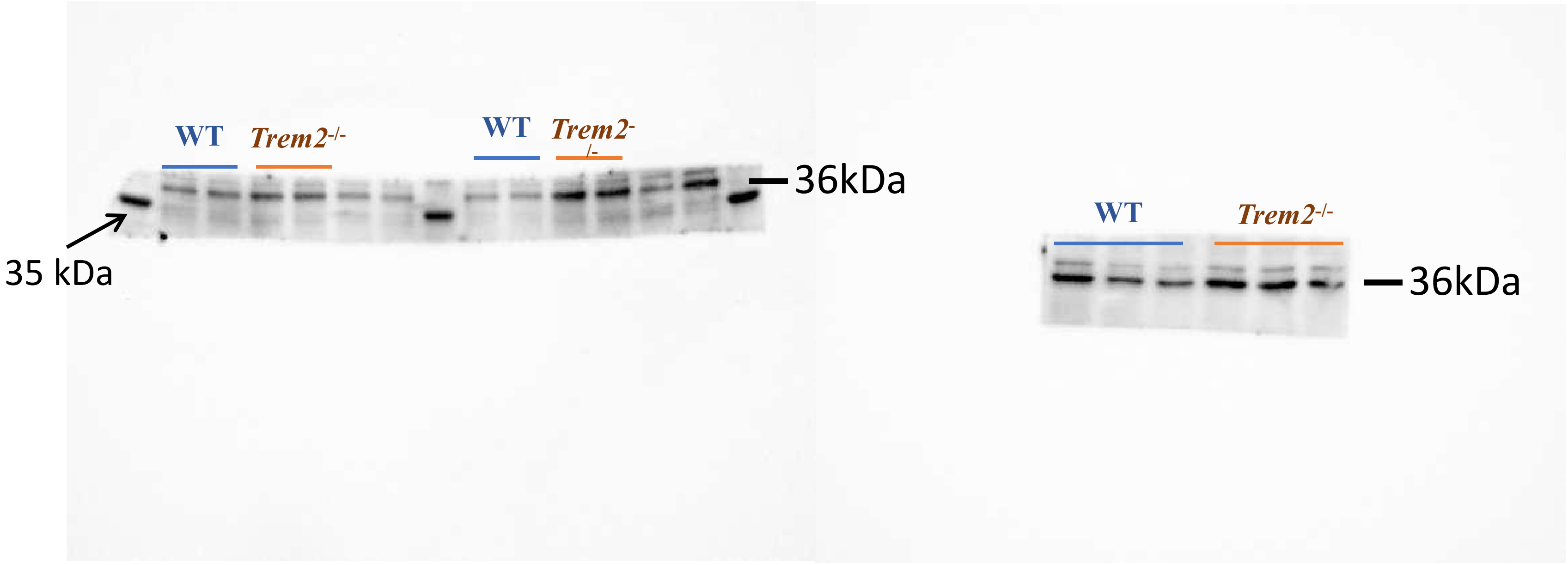

Figure 7e western blots

JAK2

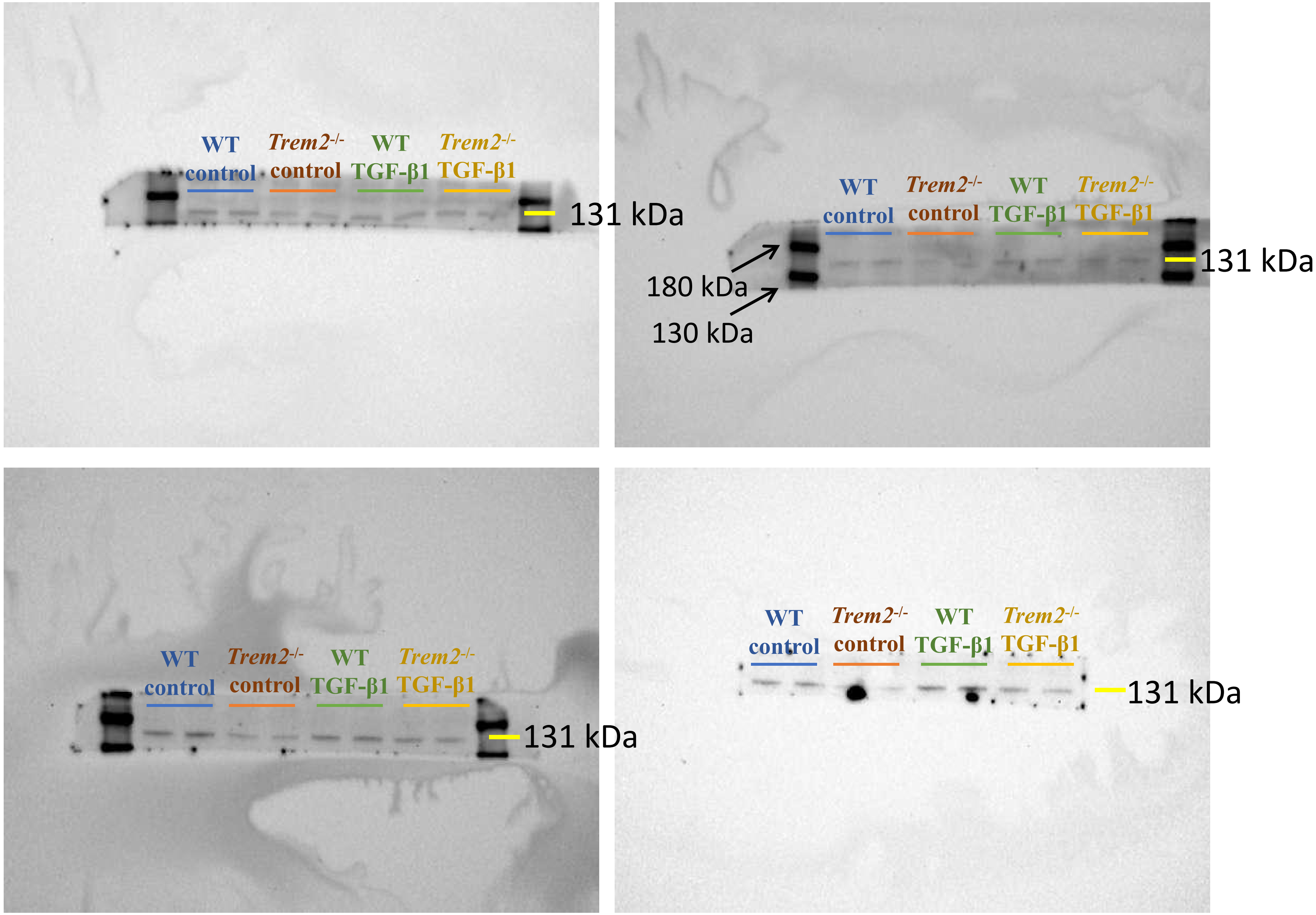

Figure 7e western blots

p-JAK2

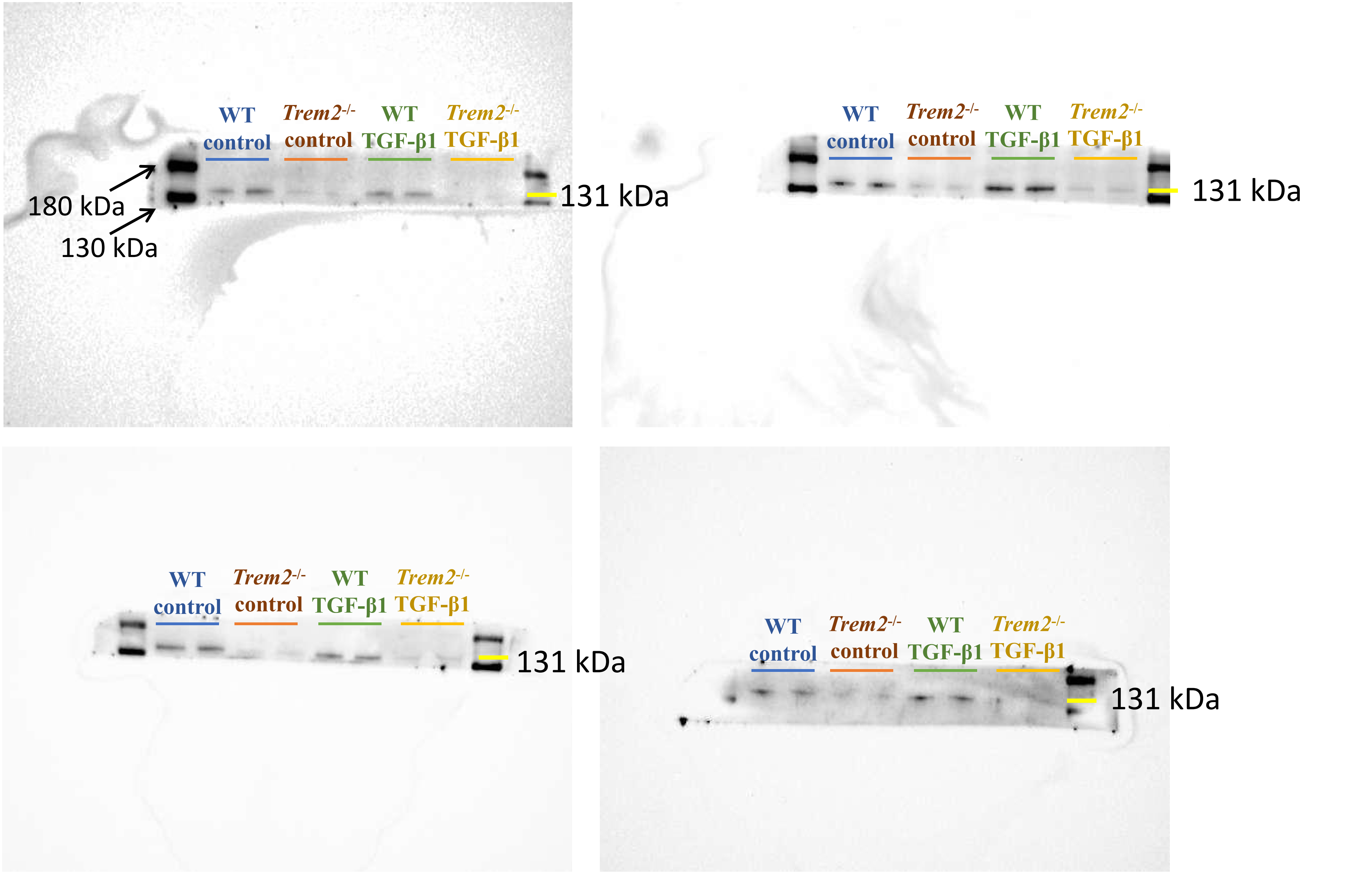

Figure 7e western blots

STAT1

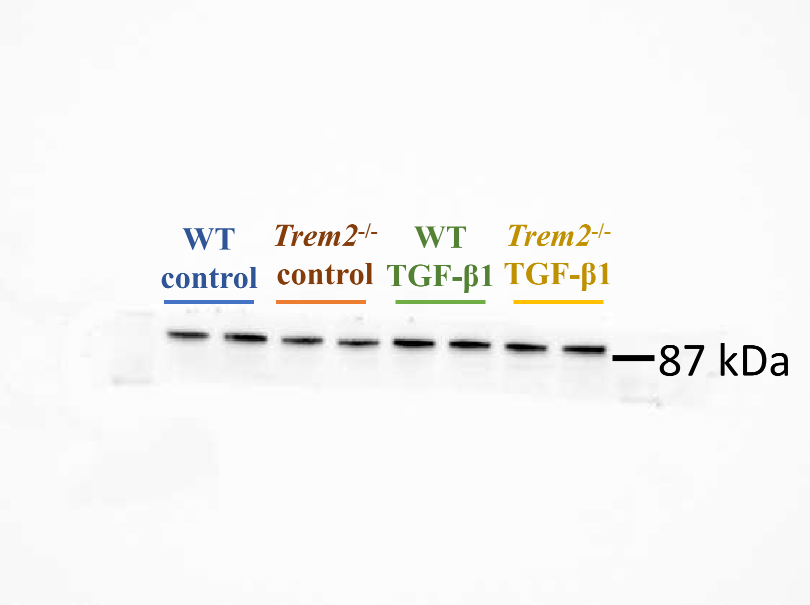

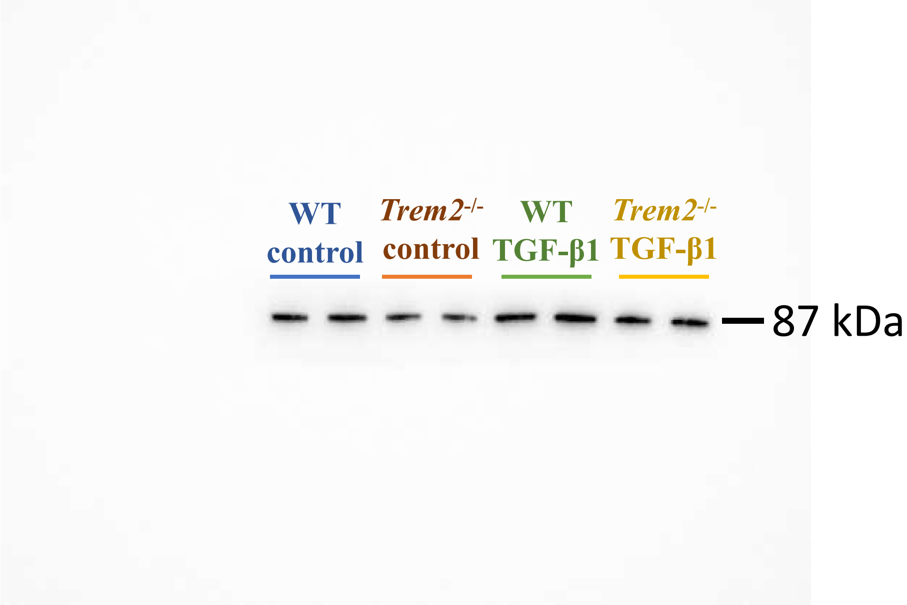

p-STAT1

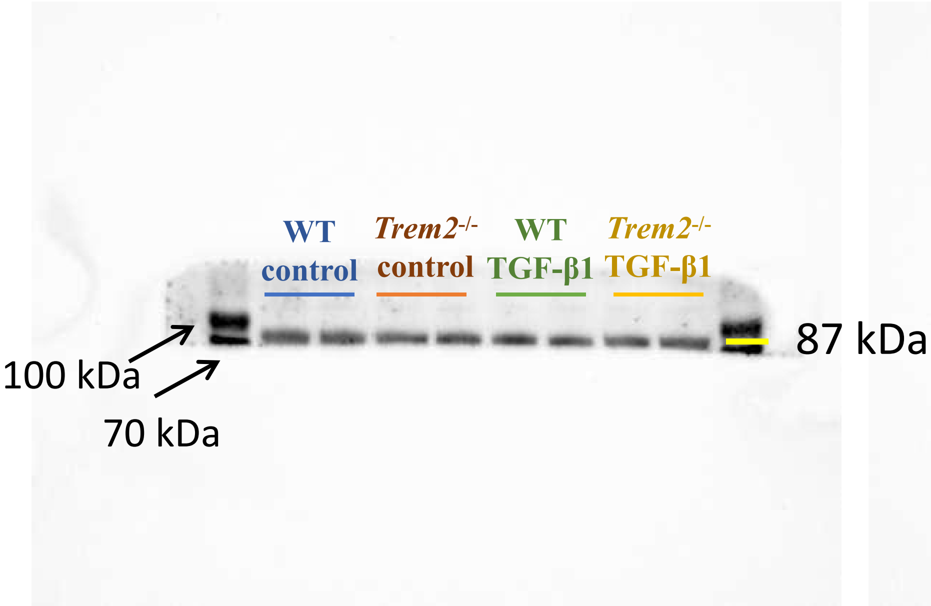

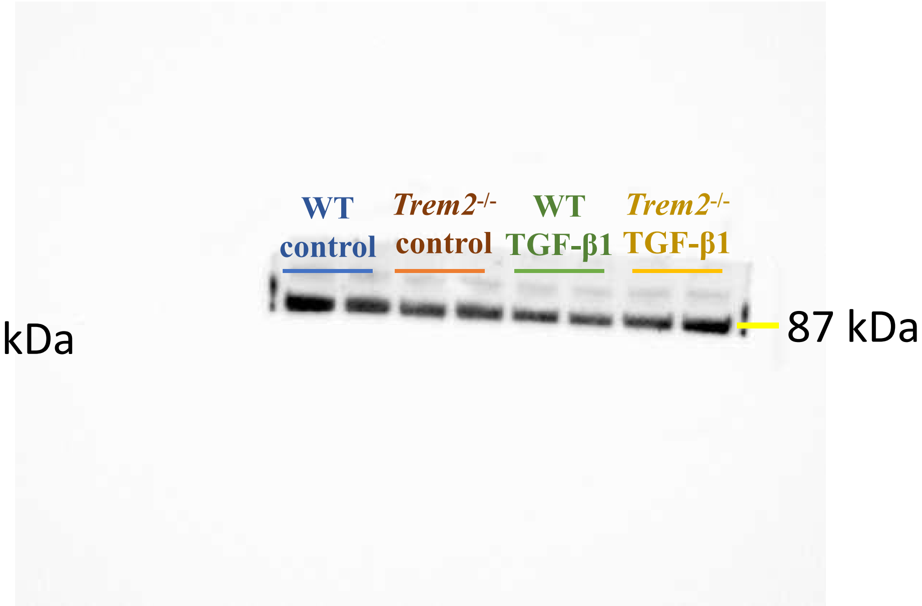

Figure 7e western blots

STAT3

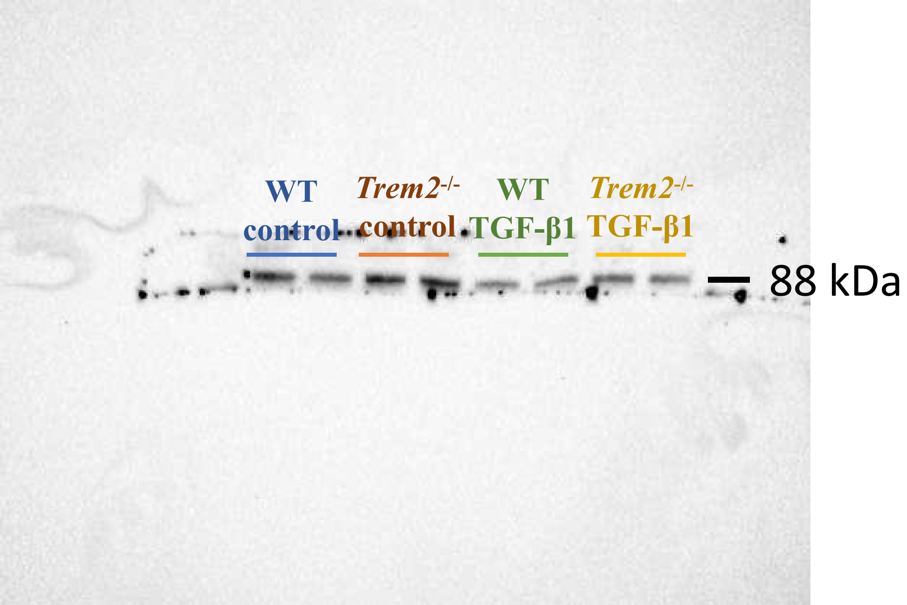

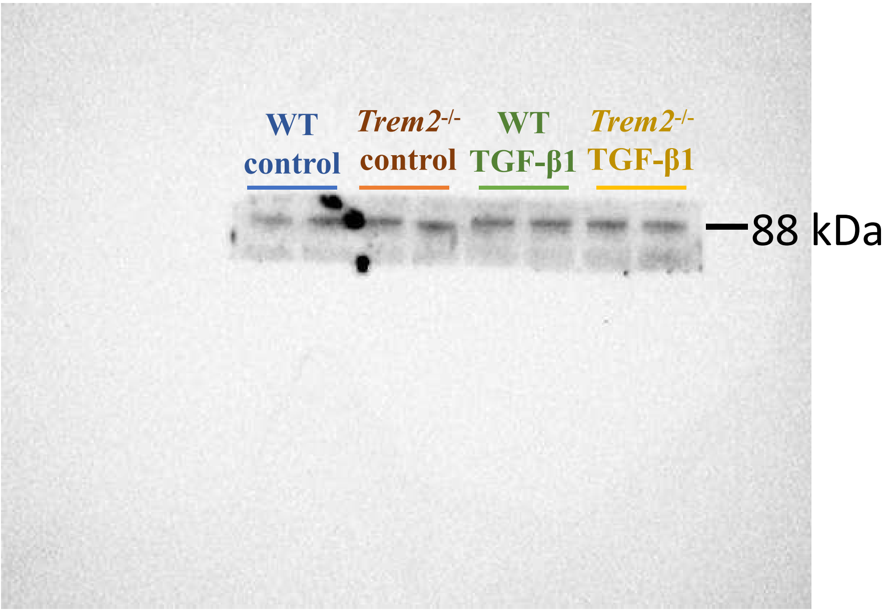

p-STAT3

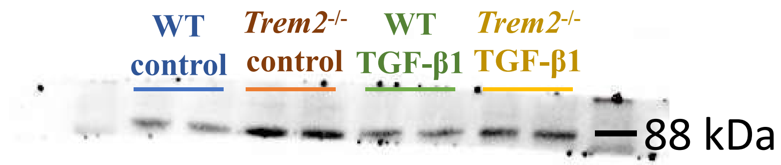

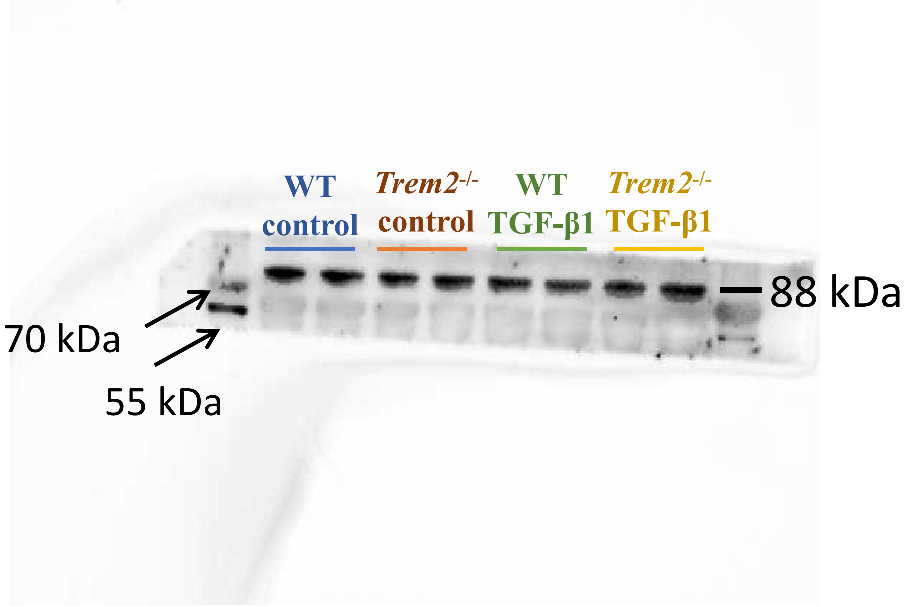

Figure 7e western blots

β-actin

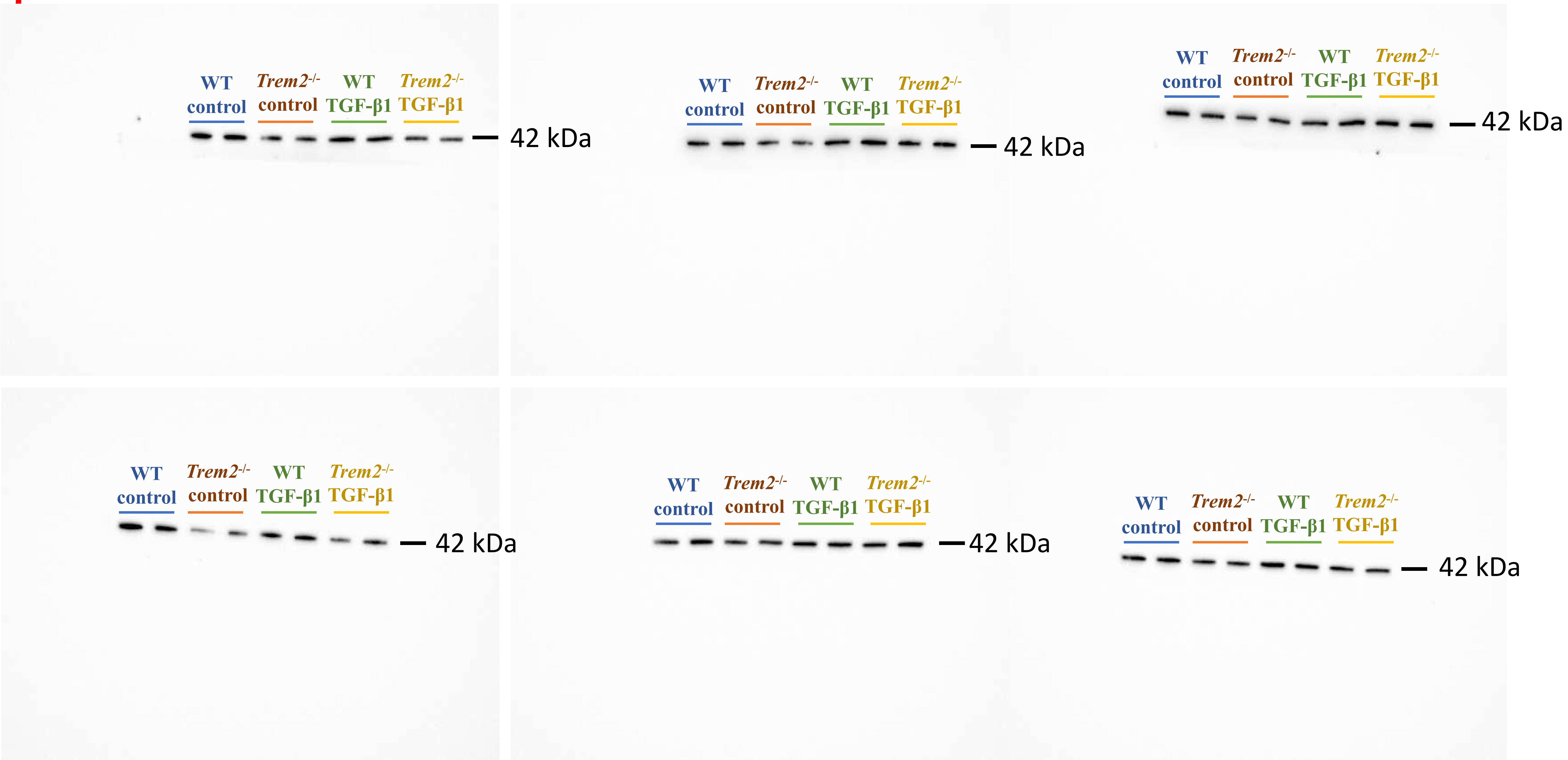

Figure 7g western blots

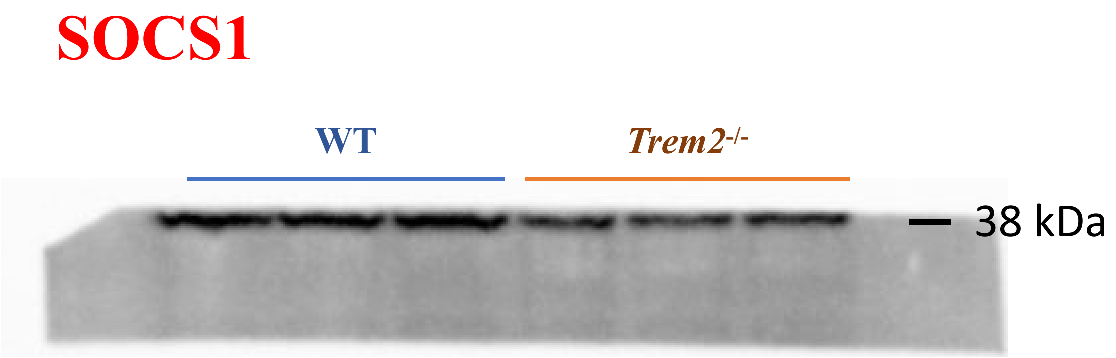

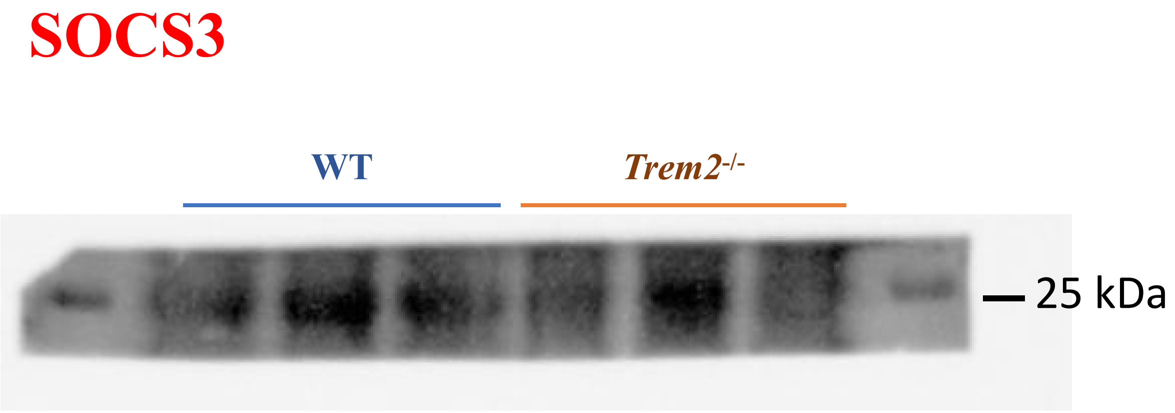

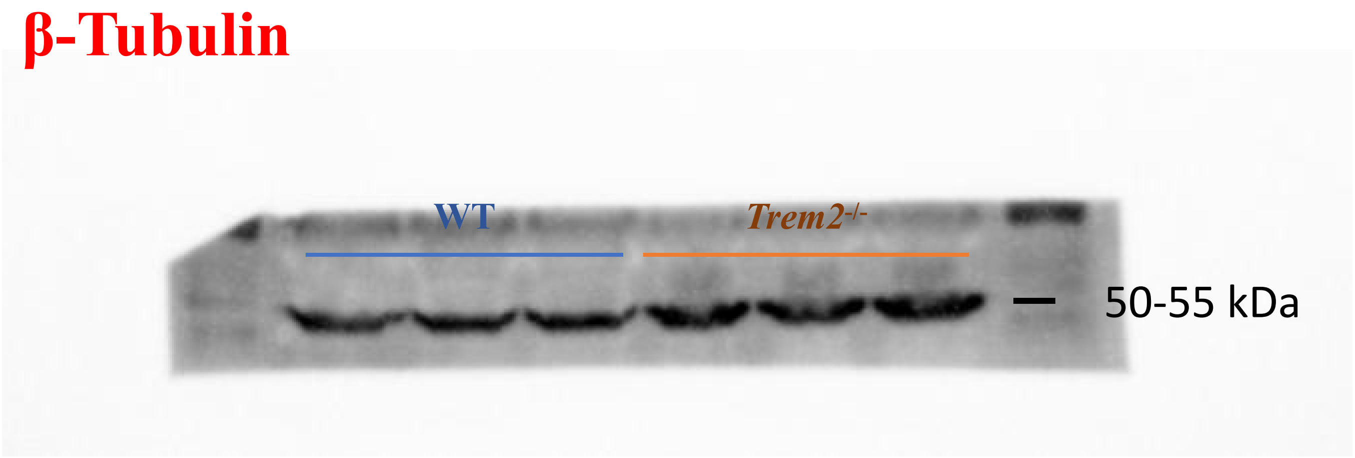

Supplement: Supplementary file 2 — Original western blots [file 41419_2024_6756_MOESM2_ESM.pdf]
